# Nonconvulsive and Electrographic Status Epilepticus in Comatose Adult Survivors of Cardiac Arrest: A Systematic Review and Meta-Analysis of Prevalence, EEG-Based Diagnosis, Treatment, and Neurological Outcomes

**DOI:** 10.3390/brainsci16090994

**Published:** 2026-09-19

**Authors:** Vivek Gupta, Viswanath Vasudevan

**Affiliations:** 1 School of Medicine, Ross University, Bridgetown BB11015, Barbados; 2 Department of Medicine, The Brooklyn Hospital Center, Brooklyn, NY 11201, USA; vvasudevan@tbh.org

**Keywords:** cardiac arrest, coma, continuous EEG, electrographic seizures, electrographic status epilepticus, nonconvulsive status epilepticus, post-anoxic status epilepticus, systematic review, meta-analysis

## Abstract

**Highlights:**

**What are the main findings?**

- The pooled prevalence of NCSE/ESE/PSE was 22% among EEG-monitored comatose survivors of cardiac arrest, although prevalence varied substantially across EEG definitions, timing, and monitoring strategies.
- EEG-defined status epilepticus and related electrographic abnormalities were associated with high rates of poor neurological outcome and death, but recovery was documented in selected patients.

**What are the implications of the main findings?**

- EEG abnormalities after cardiac arrest should not be interpreted as a uniform marker of futility; prognosis should be based on multimodal assessment, including EEG background, timing of onset, and other poor-outcome markers.
- Future studies should use standardized EEG definitions, consistent denominators, and phenotype-enriched treatment designs to identify patients most likely to benefit from active intervention.

**Abstract:**

**Objectives**: This systematic review and meta-analysis aimed to estimate the prevalence of nonconvulsive status epilepticus (NCSE), electrographic status epilepticus (ESE), post-anoxic status epilepticus (PSE), and electrographic seizures in comatose adult survivors of cardiac arrest and to summarize associated diagnostic approaches, treatment strategies, neurological outcomes, and mortality. **Methods**: PubMed/MEDLINE, MEDLINE Core, Cochrane CENTRAL, Web of Science, and Scopus were searched from inception to May 2026. Eligible studies included adult comatose survivors of cardiac arrest who underwent EEG monitoring and reported NCSE, ESE, PSE, electrographic seizures, or related rhythmic/periodic EEG patterns. Random-effects meta-analysis of proportions was performed, separating strict NCSE/ESE/PSE constructs from broader electrographic seizure/status and epileptiform/IIC constructs. **Results**: Seventeen studies were included. In the primary analysis of four non-overlapping EEG-monitored cohorts, strict NCSE/ESE/PSE occurred in 112 of 457 patients; the pooled prevalence was 22% (95% CI, 10–41%; 95% prediction interval, 4–64%; I^2^ = 77.8%). The expanded sensitivity analysis including selected, historical, or restricted cohorts yielded a similar pooled prevalence of 22% (95% CI, 16–31%). Broad electrographic seizure/status prevalence was 10% (95% CI, 2–33%; I^2^ = 94.7%), and broad epileptiform/ictal–interictal continuum pattern prevalence was 29% (95% CI, 7–70%; I^2^ = 86.0%); these analyses represented distinct EEG constructs. Poor neurological outcome and mortality were frequent, with pooled estimates of 91% (95% CI, 85–94%) and 87% (95% CI, 78–93%), respectively. **Conclusions**: The pooled prevalence of NCSE/ESE/PSE was 22% among EEG-monitored comatose survivors of cardiac arrest, although the exact prevalence remains uncertain across settings. Poor neurological outcome and mortality were frequent, but recovery occurred in selected patients with continuous EEG background, later SE onset, fewer multimodal poor-outcome markers, or electrographic cessation. These findings support multimodal, phenotype-guided interpretation rather than reliance on EEG status alone. Future studies should use standardized EEG definitions, consistent denominators, and phenotype-enriched randomized designs.

## 1. Introduction

Neurological injury is a major determinant of outcome among adults who remain comatose after cardiac arrest, and electroencephalography (EEG) is central to post-resuscitation neurological assessment [1]. Current post-resuscitation and neurocritical care guidance supports continuous EEG (cEEG) monitoring to screen for seizures or status epilepticus (SE) in patients who fail to recover consciousness, with daily intermittent EEG during the first 72–120 h when cEEG is unavailable [2,3].

Although EEG enables detection of electrographic seizure activity that may be clinically occult, its interpretation after cardiac arrest remains challenging. Nonconvulsive status epilepticus (NCSE), electrographic status epilepticus (ESE), post-anoxic status epilepticus (PSE), electrographic seizures, and ictal–interictal continuum (IIC) patterns are difficult to classify because coma, sedation, neuromuscular blockade, and temperature management may obscure clinical manifestations [4]. The American Clinical Neurophysiology Society (ACNS) terminology and Salzburg criteria have improved standardization of electrographic seizure, ESE, and NCSE classification; however, their application across post-cardiac arrest studies remains heterogeneous with respect to EEG modalities, monitoring durations, timing of EEG initiation, and diagnostic thresholds [5,6]. Consequently, reported frequencies have varied substantially, with rhythmic or periodic EEG patterns reported in 10–35% of comatose patients after cardiac arrest, ESE reported in 12–31% in earlier cEEG cohorts, and NCSE reported in 1–20% in prior focused review evidence [7,8,9].

The prognostic interpretation of these EEG-defined entities remains clinically important but uncertain. Post-anoxic SE has generally been associated with high mortality and poor neurological outcome; however, recovery has been reported in selected patients, particularly when SE develops after recovery of a continuous EEG background and in the absence of multiple poor-outcome markers [7,10,11]. In recent cohorts, favorable outcome was uncommon in unselected PSE populations but appeared more frequent in patients with late-onset PSE, continuous background activity before SE onset, and favorable multimodal prognostic profiles [10,11].

This prognostic uncertainty directly complicates treatment decisions. In the TELSTAR trial, protocolized suppression of rhythmic and periodic EEG activity did not reduce poor neurological outcome at 3 months compared with standard care, despite greater electrographic suppression in the intervention group [7]. Conversely, pooled patient-level evidence suggested that SE cessation, EEG background continuity, and lower burden of poor-outcome prognostic criteria may identify subgroups in whom prolonged treatment could be associated with recovery [11]. Thus, the central therapeutic question is not simply whether electrographic abnormalities can be suppressed, but whether treatment alters neurological outcome and which electroclinical phenotypes may remain treatment-responsive. Prior reviews have primarily addressed EEG prognostication after hypoxic–ischemic brain injury or NCSE frequency after return of spontaneous circulation [12]. Accordingly, an important evidence gap remains at the intersection of EEG detection, diagnostic classification, treatment responsiveness, and neurological prognosis. The present review separately synthesized strict NCSE/ESE/PSE, broader electrographic seizure/status patterns, treatment-response evidence, and neurological outcomes while accounting for cohort overlap and denominator heterogeneity. Specifically, this systematic review addressed three key questions: (1) How frequently do strict NCSE/ESE/PSE and broader electrographic seizure/status patterns occur among EEG-monitored comatose survivors of cardiac arrest? (2) How do EEG monitoring approaches and diagnostic definitions vary across studies? and (3) What treatment-response patterns, neurological outcomes, and mortality are reported in patients with these EEG-defined conditions?

## 2. Materials and Methods

### 2.1. Protocol and Reporting Standards

This systematic review and meta-analysis was conducted in accordance with the Preferred Reporting Items for Systematic Reviews and Meta-Analyses (PRISMA) 2020 statement [13]. The completed PRISMA 2020 checklist is provided in Supplementary File S1. The review protocol was developed a priori and registered in the International Prospective Register of Systematic Reviews (PROSPERO; CRD420261402255).

### 2.2. Eligibility Criteria

Studies were eligible if they included adult patients aged ≥18 years who remained comatose after successful resuscitation from out-of-hospital or in-hospital cardiac arrest and underwent EEG assessment after return of spontaneous circulation (ROSC). Eligible EEG modalities included routine EEG, continuous EEG, amplitude-integrated EEG, reduced-montage EEG, or critical care EEG monitoring. The index conditions were EEG-confirmed NCSE, ESE, PSE, electrographic seizures, or clearly defined IIC, rhythmic, or periodic EEG patterns after cardiac arrest. Eligible study designs included prospective and retrospective cohort studies, registry-based studies, post hoc analyses of randomized or prospective studies, randomized controlled trials, pooled individual-patient or aggregate-data analyses, and case series with at least 10 eligible post-cardiac arrest patients.

Studies were excluded if they included pediatric-only populations, general intensive care or neurological intensive care cohorts without separately reported post-cardiac arrest data, convulsive status epilepticus without EEG-confirmed nonconvulsive or electrographic data, case reports or very small case series (<10 eligible patients), narrative reviews, editorials, commentaries, letters without original data, animal studies, and in vitro studies. Non-English articles were excluded unless sufficient extractable data were available from the abstract, tables, supplementary material, or reliable translation.

### 2.3. Information Sources and Search Strategy

A systematic literature search was conducted from database inception to May 2026. The following databases were searched: PubMed/MEDLINE, MEDLINE Core, Cochrane Central Register of Controlled Trials, Web of Science Core Collection, and Scopus. Reference lists of included studies and relevant reviews were manually screened. The search strategy combined terms for cardiac arrest, post-resuscitation state, ROSC, status epilepticus, nonconvulsive seizures, electrographic seizures, rhythmic and periodic EEG patterns, IIC patterns, and EEG monitoring. No date restriction was applied. The complete search strategies for each database are provided in Supplementary File S2.

### 2.4. Study Selection

All retrieved records were imported into EndNote X9, and duplicates were removed before screening. Title and abstract screening were performed independently by two reviewers (V.G and V.V) using the Rayyan web application (Rayyan Systems Inc., Cambridge, MA, USA. Full texts of potentially eligible records were then assessed independently against the prespecified eligibility criteria. Disagreements were resolved by discussion.

### 2.5. Data Extraction

Data were extracted independently by two reviewers using a standardized extraction form. Discrepancies were resolved by consensus or adjudication by a third reviewer. Extracted study characteristics included first author, publication year, country, study design, setting, study period, sample size, population, follow-up duration, reported outcomes, and brief main findings. Extracted population characteristics included age, sex, cardiac arrest location, initial rhythm, witnessed arrest, bystander cardiopulmonary resuscitation, no-flow and low-flow time, time to ROSC, cause of arrest, coma definition, sedation exposure, targeted temperature management use, temperature target, timing of temperature management, and withdrawal-of-life-sustaining-therapy policies when reported.

EEG variables included modality, timing from return of spontaneous circulation to EEG initiation, monitoring duration, diagnostic criteria, background category, reactivity, rhythmic or periodic patterns, electrographic seizure or status epilepticus onset, and study-specific definitions of NCSE, ESE, PSE, electrographic seizures, and IIC patterns. Treatment variables included antiseizure medication use, number and type of antiseizure medications, sedative or anesthetic infusions, treatment escalation, EEG-guided suppression, timing of treatment initiation, electrographic cessation, recurrence, and treatment-related complications.

### 2.6. Outcomes

The primary outcome was pooled prevalence of strict NCSE/ESE/PSE among adult comatose survivors of cardiac arrest who underwent EEG monitoring. Strict NCSE/ESE/PSE was defined as EEG-confirmed electrographic or electroclinical status epilepticus meeting study-specific criteria broadly consistent with sustained electrographic seizure activity or ACNS/Salzburg-defined definite or possible post-anoxic SE, excluding isolated non-evolving IIC or rhythmic/periodic patterns unless classified as SE by the original study. Secondary outcomes included broad electrographic seizure/status prevalence, broad epileptiform/IIC pattern prevalence, favorable neurological outcome, poor neurological outcome, survival, mortality, electrographic seizure or status epilepticus cessation, and treatment-response outcomes. Favorable neurological outcome was preferentially defined as Cerebral Performance Category (CPC) 1–2 or modified Rankin Scale (mRS) 0–3. Poor neurological outcome was defined as CPC 3–5, mRS 4–6, or the study-specific unfavorable outcome definition. When several timepoints were reported, the prespecified primary or latest clinically relevant outcome timepoint was extracted and documented.

### 2.7. Risk-of-Bias Assessment

Risk of bias was assessed independently by two reviewers according to study design. Observational, registry-based, post hoc, and descriptive studies contributing prevalence or frequency estimates were assessed using the Joanna Briggs Institute (JBI) critical appraisal checklist for studies reporting prevalence data [14]. Non-randomized studies evaluating treatment-effect or treatment-intensity evidence were assessed using Risk Of Bias In Non-randomized Studies of Interventions (ROBINS-I) [15]. Randomized studies were assessed using the Cochrane Risk of Bias (RoB 2) tool [16]. Disagreements were resolved by discussion or adjudication by a third reviewer. Risk-of-bias judgements informed interpretation, sensitivity analyses, and certainty assessment but were not used as automatic exclusion criteria. Certainty of evidence was assessed using an adapted GRADE approach at the outcome level, considering risk of bias, inconsistency, indirectness, imprecision, and publication bias. For prevalence and absolute outcome-frequency estimates, observational evidence was initially rated as high certainty because observational cohorts represent the appropriate design for estimating event rates, and was subsequently downgraded according to the standard GRADE domains. Randomized treatment evidence started at high certainty, whereas non-randomized treatment-effect evidence started at low certainty [17].

### 2.8. Data Synthesis and Statistical Analysis

A narrative synthesis was performed for all included studies. Meta-analysis was conducted when studies were sufficiently comparable in population, EEG definition, denominator, outcome definition, and follow-up timepoint. For single-arm prevalence outcomes, event counts and denominators were extracted at the patient level whenever possible. The denominator was preferentially defined as the number of adult comatose post-cardiac arrest patients who underwent EEG monitoring. If all eligible post-cardiac arrest patients underwent EEG, the full cohort denominator was used. If only a selected subgroup underwent EEG, the monitored subgroup was used as the denominator, and selection criteria were recorded. Random-effects models were used because clinical and methodological heterogeneity was expected across studies. Proportions were pooled using a logit-transformed random-effects model with restricted maximum likelihood and Hartung–Knapp adjustment. Freeman–Tukey double-arcsine transformation was considered during statistical planning for analyses with very low or very high prevalence estimates but was not applied because the final prevalence analyses were adequately handled using the logit-transformed model.

Secondary meta-analyses were conducted for poor neurological outcome, mortality, and comparative associations where data permitted. For dichotomous comparative outcomes, pooled odds ratios with 95% confidence intervals were calculated and interpreted as exploratory when fewer than three studies contributed data. Adjusted estimates were preferred over unadjusted estimates when clinically appropriate and sufficiently reported. Where overlapping cohorts were identified, the study with the largest sample size, longest follow-up, most complete EEG data, or most relevant outcome reporting was prioritized for each analysis to avoid double counting. If one publication provided prevalence data and another provided more detailed EEG phenotype or treatment-outcome data from the same cohort, each was used only for the non-overlapping endpoint for which it was most informative. Statistical heterogeneity was assessed using the I^2^ statistic, τ^2^, and visual inspection of forest plots. Prediction intervals were calculated when feasible. Publication bias and small-study effects were not assessed when fewer than 10 studies contributed to a meta-analysis. Analyses were performed using R software version 4.6.0 for Windows, using the meta package version 8.5-0 and metafor package version 5.0-1; dplyr, readr, ggplot2, and stringr were used for data handling and figure preparation. A two-sided *p* value < 0.05 was considered statistically significant where hypothesis testing was performed.

## 3. Results

### 3.1. Study Selection

**Figure 1:**
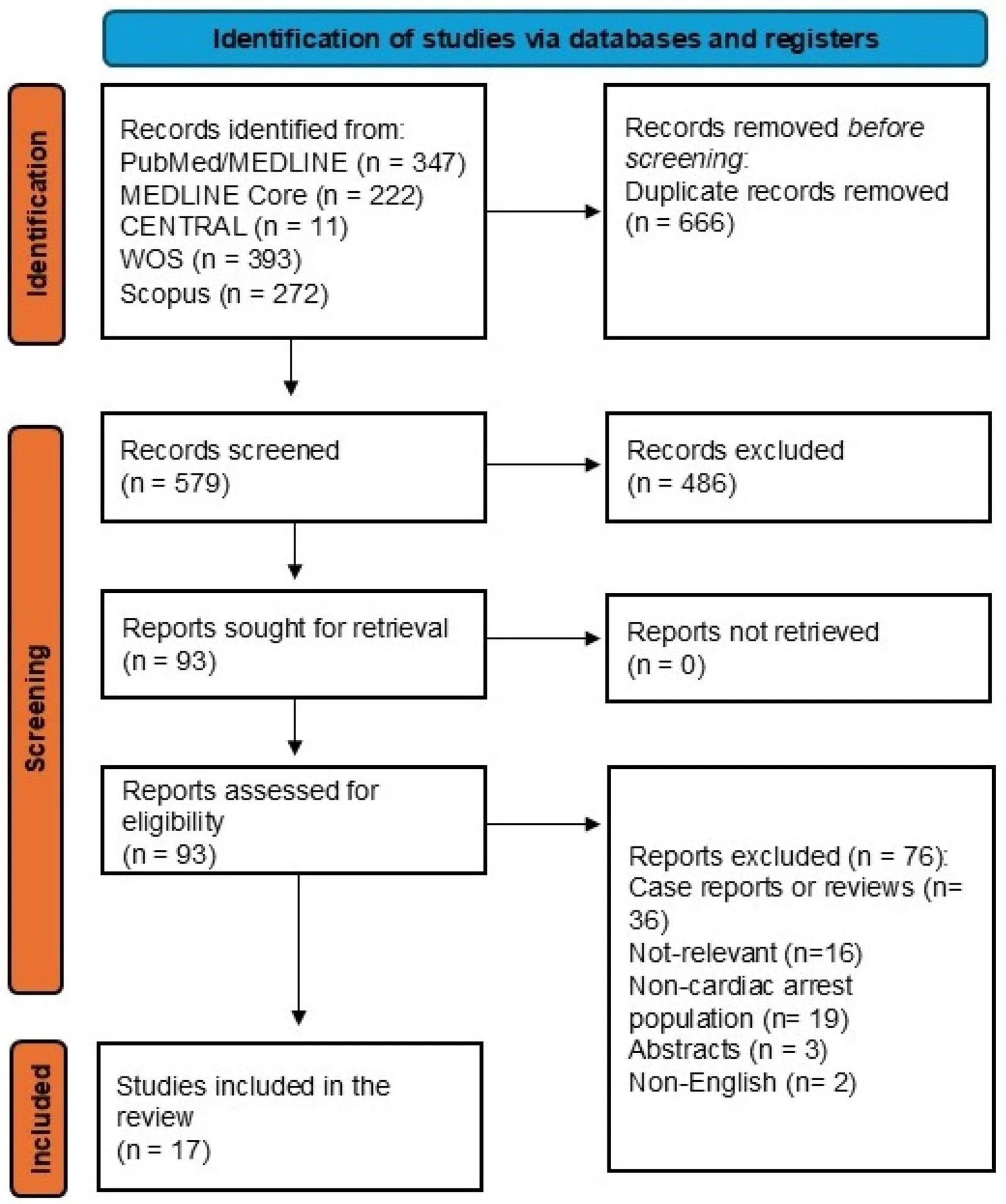
PRISMA 2020 flow diagram of study selection.

The database searches identified 1245 records, including 347 from PubMed/MEDLINE, 222 from MEDLINE Core, 11 from Cochrane CENTRAL, 393 from Web of Science, and 272 from Scopus. After removal of 666 duplicates, 579 records underwent title and abstract screening. Ninety-three reports were assessed in full text, of which 17 studies met the eligibility criteria and were included in the systematic review [7,8,10,11,18,19,20,21,22,23,24,25,26,27,28,29,30]. The study selection process is summarized in the PRISMA flow diagram (Figure 1).

### 3.2. Study Characteristics

Across the included evidence base, 17 studies were identified from Europe and the United States, comprising retrospective registry or cohort studies, prospective/post hoc trial substudies, one randomized treatment trial, one randomized EEG-monitoring strategy analysis, and one pooled individual-patient data analysis. Study populations varied substantially, ranging from small single-center cEEG cohorts [e.g., Mani et al. [24], *N* = 38] to large post-arrest EEG cohorts [e.g., Faro et al. [22], *N* = 818], with follow-up assessed from hospital discharge to 24 months. Four studies were considered suitable for primary strict NCSE/ESE/PSE prevalence estimation, whereas others were assigned to restricted or supplementary analytical roles because of selected denominators, cohort overlap, treatment-based inclusion, phenotype-only sampling, or broader electrographic/IIC definitions. Overall, the studies showed that NCSE/ESE/PSE and related electrographic patterns were variably frequent after cardiac arrest, with strict ESE/PSE estimates ranging from 11.9% to 32.3% in the primary prevalence-eligible cohorts, while outcomes were generally poor, particularly in patients with early status epilepticus, malignant EEG background, or absence of favorable prognostic profiles. Several studies also suggested clinically relevant heterogeneity: continuous or non-suppressed EEG background, later-onset SE, SE cessation, and absence of multiple poor-outcome prognostic markers were associated with potential recovery in selected cohorts, whereas protocolized EEG suppression in the TELSTAR trial increased electrographic suppression without reducing poor neurological outcome or mortality (Table 1).

### 3.3. Patient Characteristics

Across studies, the median or mean age generally ranged from 57 to 70 years, although younger patients were represented in the substance-use-related cardiac arrest cohort (49 ± 12 years). Men constituted the majority of most cohorts, ranging from 52.6% in Mani et al. to 81.8% in the substance-use-related subgroup of Shih et al. [24,30]. Where reported, out-of-hospital cardiac arrest accounted for most enrolled cases, including 82.6% in Pelle et al. [21], 85.0% in Dragancea et al., 89.1% in De Stefano et al. [11], and 100% in the Admiraal et al. [10,27] and Shih et al. cohorts [30]. The proportion of patients with shockable rhythm varied substantially across studies, from 11.2% in substance-use-related cardiac arrest to 71.6% in the TELSTAR control arm, reflecting marked differences in arrest etiology, cohort selection, and eligibility criteria. Time to ROSC, when available, was typically reported as a median of approximately 16–34 min. Targeted temperature management was used in most contemporary cohorts, although temperature targets differed across studies, including 33 °C hypothermia, 36 °C targeted management, TTM2 randomization to hypothermia or normothermia with fever control, and institution-specific protocols (Table 2).

### 3.4. Risk of Bias

Risk-of-bias assessments indicated substantial methodological heterogeneity across the included evidence base. Among observational studies assessed using the JBI prevalence checklist, seven studies were judged as having moderate concern and six as having serious concern. The main limitations were selected or restricted denominators, variation in EEG monitoring strategy, small sample size in some cohorts, older or broad diagnostic criteria, clinically abstracted EEG data without central rereview in some studies, and residual confounding by baseline hypoxic–ischemic injury severity and withdrawal of life-sustaining therapy. The primary prevalence cohorts by Dragancea et al. and Rittenberger et al. were judged as having moderate concern, reflecting consecutive cohort designs and cEEG or simplified cEEG monitoring, although outcome interpretation remained vulnerable to confounding by illness severity and WLST decisions [8,29]. Studies with selected denominators or restricted populations, including Peluso et al., Faro et al., and Rossetti et al., were judged as serious for prevalence or frequency estimation and were retained for restricted, sensitivity, or descriptive analyses [18,22,28]. Non-randomized treatment-effect evidence was assessed using ROBINS-I; Hofmeijer et al. was judged as serious/critical [23], and De Stefano et al. was judged as serious for treatment-effect inference because treatment intensity and treatment response were strongly confounded by indication and cohort overlap [11]. Randomized evidence was assessed using RoB 2; the TELSTAR trial had some concerns because the intervention was open-label despite blinded endpoint assessment [7], and Urbano et al. had some concerns/moderate concern because the post-cardiac arrest analysis was underpowered for clinical outcome differences [25]. No study was excluded solely on the basis of risk of bias. Detailed study-level risk-of-bias assessments are provided in Supplementary File S3.

### 3.5. EEG Monitoring and Diagnostic Definitions

EEG acquisition and diagnostic classification varied substantially across studies. Continuous EEG was used in several cohorts, including simplified 2-channel cEEG in the Lund/Skåne studies, 22-channel cEEG in Rittenberger et al., 19-channel cEEG in Hofmeijer et al., and multichannel cEEG in the TTM2 and TELSTAR-related studies, whereas other studies used intermittent EEG, repeated routine EEG, or mixed center-specific monitoring strategies [7,10,23,27,29]. EEG initiation ranged from early monitoring within the first 7–15 h after cardiac arrest in studies such as Rittenberger et al. [29], Mani et al. [24], and Admiraal et al. [10,27] to later first EEG assessment at a median of 2 days in Pelle et al. and 46 h in Orav et al. [19,21]. Monitoring duration also differed markedly, from 20 min EEG recordings in Pelle et al. and a minimum of 20 min intermittent EEG in Orav et al. to continuous recordings for 48–72 h or up to 5 days in cEEG-based studies [19,21]. Diagnostic definitions included ACNS terminology, Salzburg criteria, Westhall classification, and study-specific operational criteria, with some studies restricted to strict NCSE/ESE/PSE definitions and others reporting broader rhythmic/periodic or IIC patterns. Blinded EEG review was reported in several key cohorts, including Pelle et al. [21], Backman et al. [20], Dragancea et al. [8], Mani et al. [24], Admiraal et al. [10,27], Lybeck et al. [26], and De Stefano et al. [11]; however, blinding was unclear or not reported in some observational or clinically abstracted datasets. This methodological heterogeneity supported the prespecified separation of strict NCSE/ESE/PSE prevalence analyses from broader electrographic seizure, rhythmic/periodic pattern, and SE-selected phenotype analyses (Table 3).

### 3.6. Primary Prevalence of Strict NCSE/ESE/PSE

**Figure 2:**
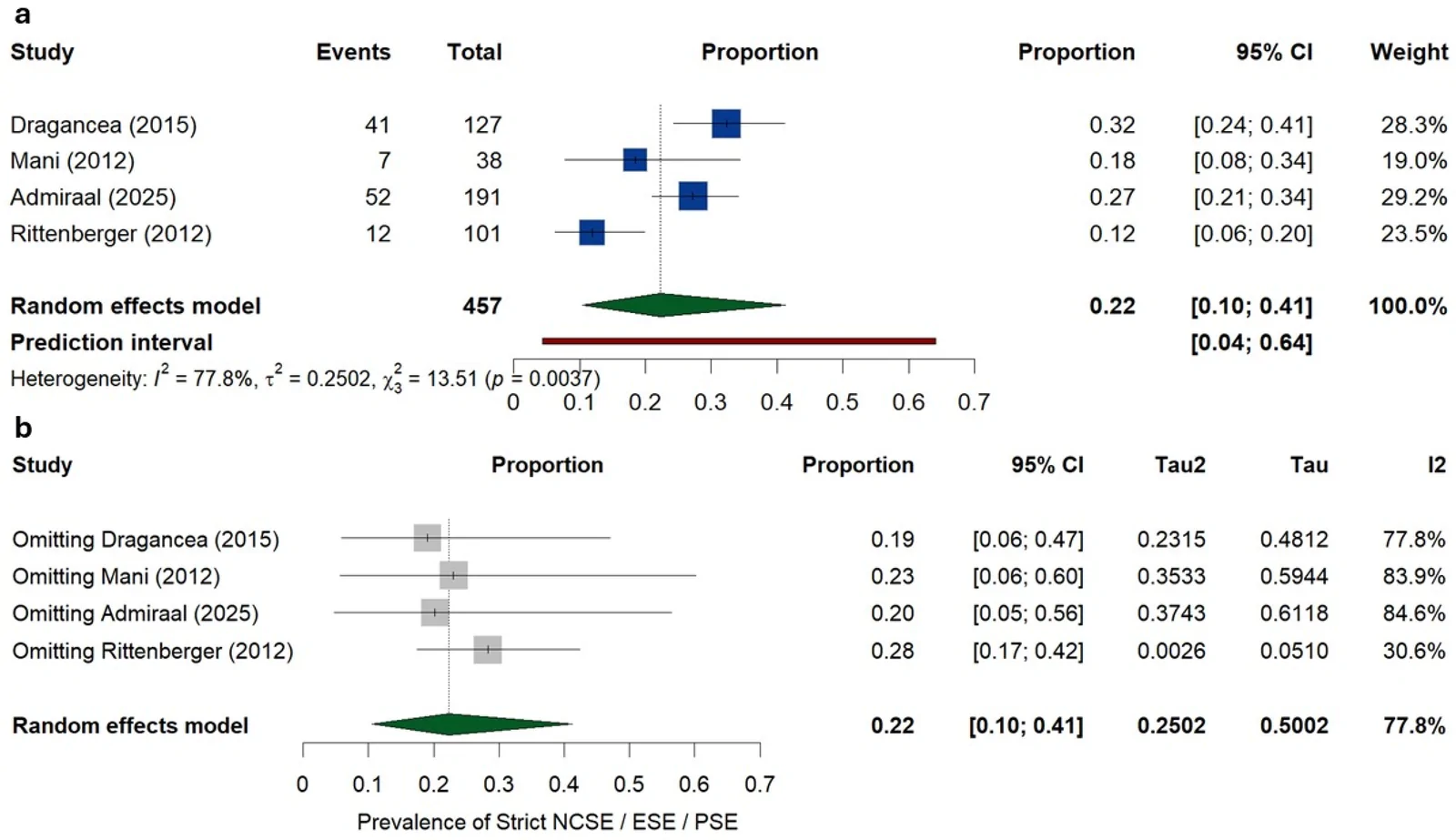
Primary prevalence of strict NCSE/ESE/PSE in non-overlapping EEG-monitored post-cardiac arrest cohorts. (**a**) Forest plot showing the pooled prevalence of strict nonconvulsive status epilepticus, electrographic status epilepticus, or post-anoxic status epilepticus among adult comatose survivors of cardiac arrest who underwent EEG monitoring. (**b**) Leave-one-out sensitivity analysis showing the influence of sequential omission of each study on the pooled prevalence estimate and heterogeneity [8,10,24,29]. For Mani et al., the strict prevalence analysis included the 7/38 patients who met the study-defined criterion for status epilepticus; the broader electrographic seizure group (9/38) was not used for this strict prevalence outcome.

The primary prevalence meta-analysis included four non-overlapping EEG-monitored post-cardiac arrest cohorts [8,10,24,29]. Strict NCSE/ESE/PSE was reported in 112 of 457 patients. The pooled prevalence was 22% (95% CI, 10–41%), with substantial between-study heterogeneity (I^2^ = 77.8%; τ^2^ = 0.2502; Q = 13.51; *p* = 0.0037) and a wide 95% prediction interval (4–64%) (Figure 2a). Study-level estimates ranged from 12% in Rittenberger et al. to 32% in Dragancea et al. [8,29]. Leave-one-out analyses showed that the pooled estimate remained between 19% and 28% after sequential omission of each study. Heterogeneity remained high after omission of Dragancea et al. [8], Mani et al. [24], or Admiraal et al. [10] but decreased to 30.6% after omission of Rittenberger et al. [29], with a pooled estimate of 28% (95% CI, 17–42%), as shown in Figure 2b. The study-level characteristics and prevalence data underlying the quantitative analyses are provided in Supplementary File S4 (worksheets: Characteristics and Prevalence).

### 3.7. Sensitivity and Exploratory Prevalence Analyses

**Figure 3:**
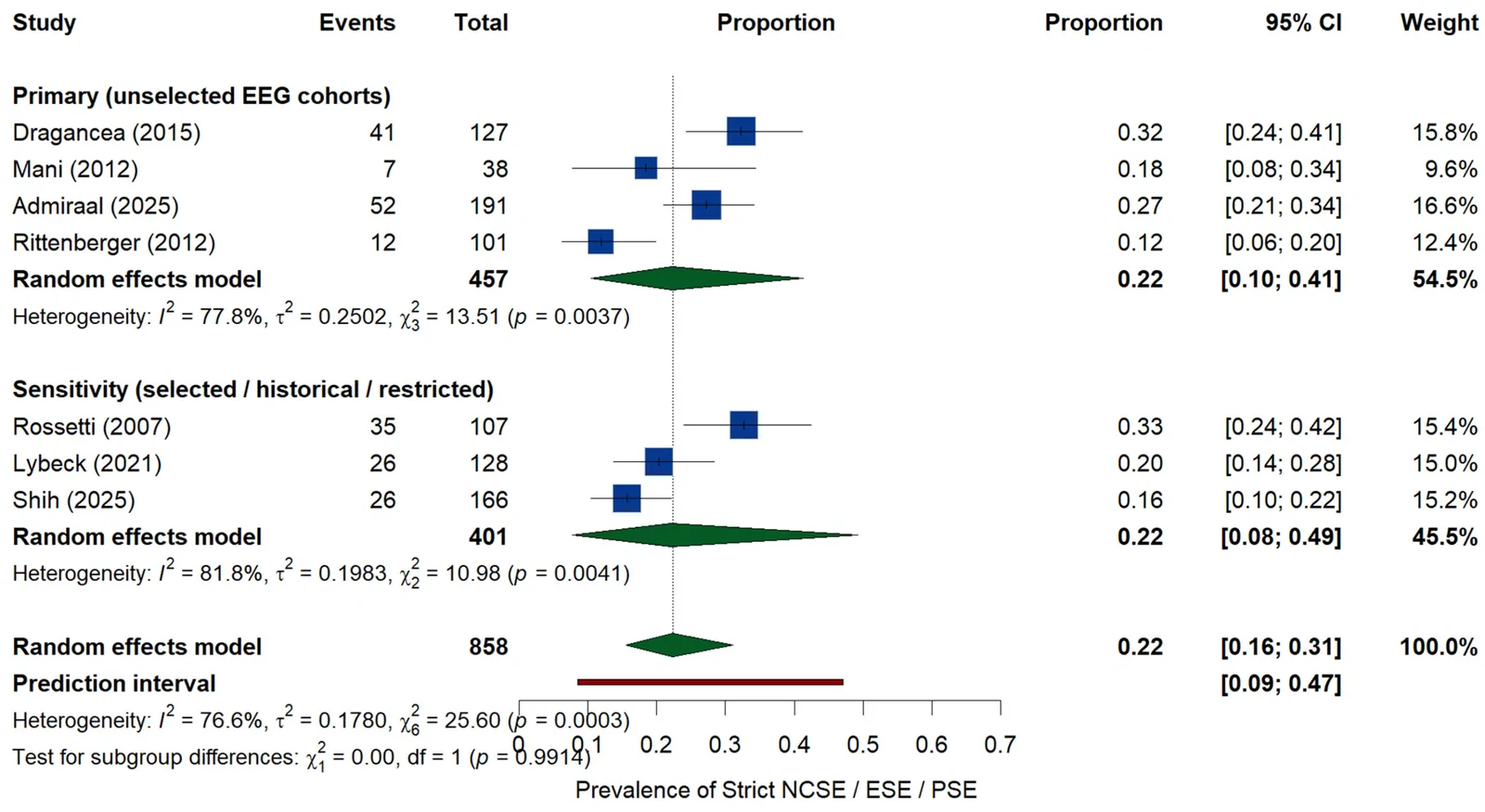
Sensitivity analysis of strict NCSE/ESE/PSE prevalence according to denominator type. Forest plot showing pooled prevalence estimates after inclusion of both primary unselected EEG-monitored cohorts and selected, historical, or restricted cohorts [8,10,24,26,28,29,30].

Sensitivity analyses were used to assess whether the primary strict prevalence estimate remained consistent when cohorts with less generalizable denominators were incorporated. When selected, historical, or restricted cohorts were added to the strict prevalence analysis, seven studies including 199 events among 858 patients were available [8,10,24,26,28,29,30]. The pooled prevalence remained 22% (95% CI, 16–31%), with substantial heterogeneity (I^2^ = 76.6%; τ^2^ = 0.1780; Q = 25.60; *p* = 0.0003) and a 95% prediction interval of 9–47%. The pooled estimate was 22% (95% CI, 10–41%) in unselected EEG-monitored cohorts and 22% (95% CI, 8–49%) in selected, historical, or restricted cohorts, with no evidence of a subgroup difference by denominator type (*p* = 0.9914) (Figure 3).

**Figure 4:**
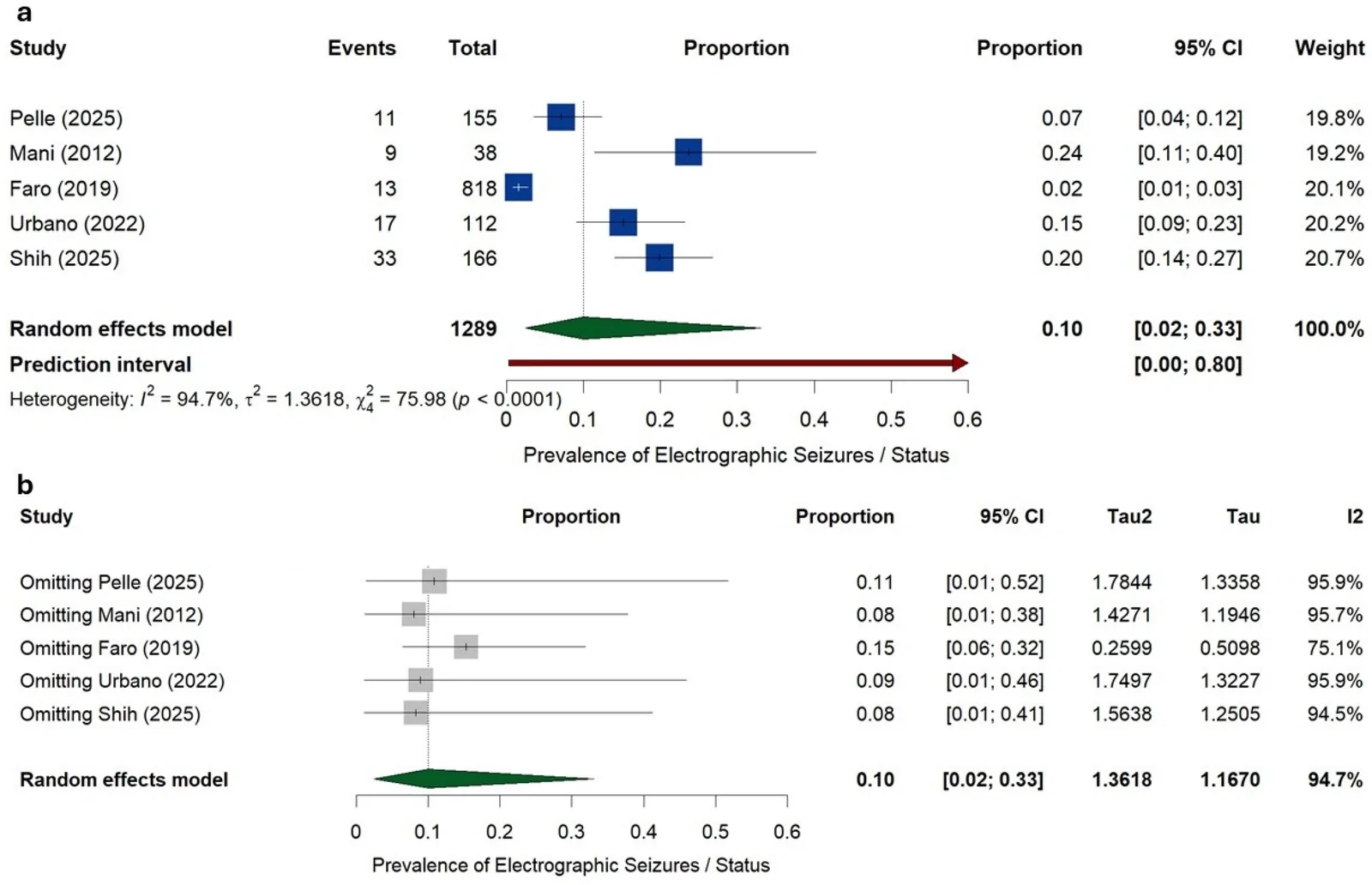
Broad electrographic seizure/status prevalence and leave-one-out sensitivity analysis. (**a**) Forest plot showing the pooled prevalence of broadly defined electrographic seizures or electrographic status among adult comatose survivors of cardiac arrest [21,22,24,25,30]. (**b**) Leave-one-out sensitivity analysis showing the effect of sequential omission of each study on the pooled estimate and heterogeneity [21,22,24,25,30].

**Figure 5:**
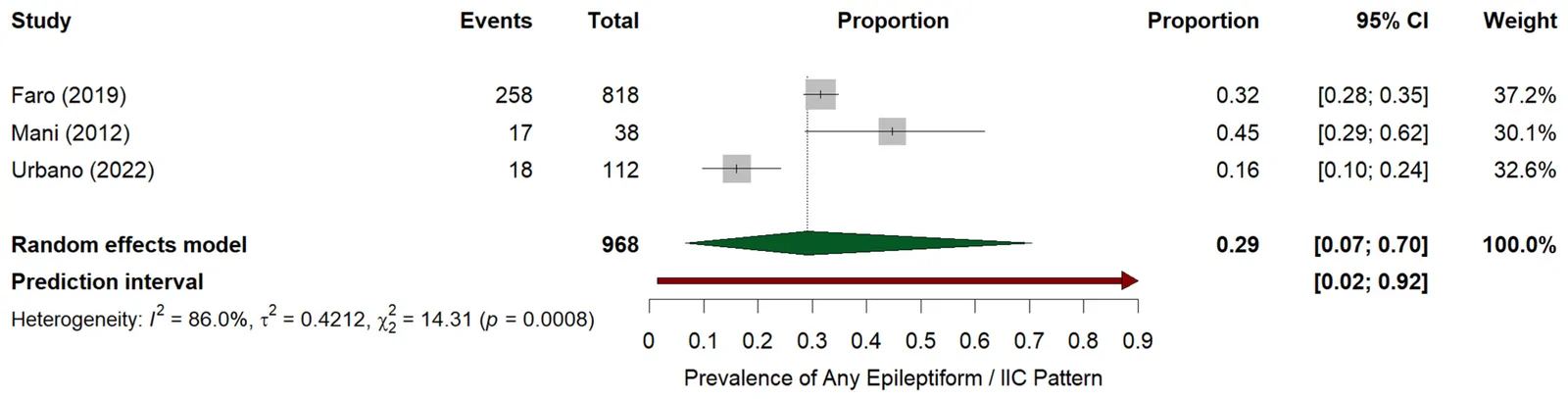
Broad epileptiform or ictal–interictal continuum pattern prevalence. Forest plot showing the pooled prevalence of broadly defined epileptiform or ictal–interictal continuum patterns after cardiac arrest [22,24,25].

Broad electrographic seizure/status prevalence was assessed separately because the included constructs were not equivalent to strict NCSE/ESE/PSE. These analyses were intended to characterize the wider spectrum of electrographic seizure-related abnormalities rather than to provide alternative estimates of the same outcome. Across five studies, electrographic seizures or status were reported in 83 of 1289 patients [21,22,24,25,30]. The pooled estimate was 10% (95% CI, 2–33%), with very high heterogeneity (I^2^ = 94.7%; τ^2^ = 1.3618; Q = 75.98; *p* < 0.0001) and a wide prediction interval (0–80%) (Figure 4a). Leave-one-out analyses showed pooled estimates ranging from 8% to 15%, with persistent heterogeneity except after omission of Faro et al. [22], which reduced I^2^ to 75.1% (Figure 4b). Broad epileptiform or IIC pattern prevalence was assessed in three studies, including 293 events among 968 patients [22,24,25]. The pooled prevalence was 29% (95% CI, 7–70%), with high heterogeneity (I^2^ = 86.0%; τ^2^ = 0.4212; Q = 14.31; *p* = 0.0008) and a 95% prediction interval of 2–92% (Figure 5). Accordingly, the broad electrographic seizure/status and epileptiform/IIC estimates should be interpreted as exploratory summaries of distinct EEG constructs, particularly given the very high heterogeneity and wide prediction intervals.

### 3.8. Conditional SE Phenotype Distributions

**Figure 6:**
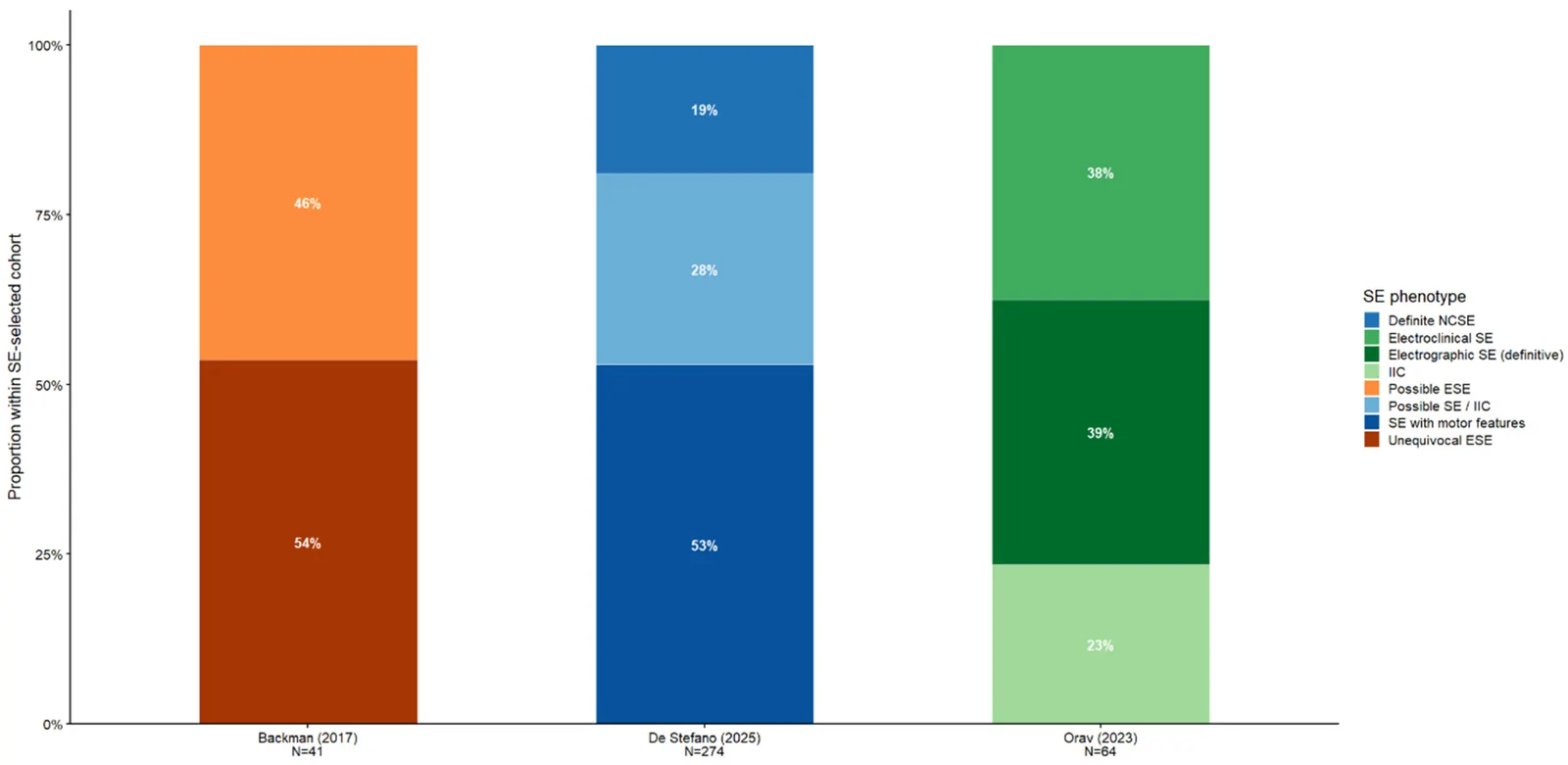
Conditional distribution of status epilepticus phenotypes within SE-selected cohorts; Stacked bar chart showing the distribution of reported SE phenotypes among patients already classified as having SE, possible SE, or related post-hypoxic EEG-defined conditions in three SE-selected cohorts [11,19,20].

Three SE-selected cohorts reported conditional phenotype distributions within patients already classified as having SE or possible SE; these proportions were therefore not interpreted as post-cardiac arrest population prevalence. In Backman et al., 54% of patients with ESE were classified as having unequivocal ESE and 46% as having possible ESE [20]. In De Stefano et al., 53% were classified as definite NCSE, 28% as possible SE/IIC, and 19% as SE with motor features [11]. In Orav et al. [19], 39% had definitive electrographic SE, 38% had electroclinical SE, and 23% had IIC. Because phenotype definitions differed across cohorts, these conditional distributions were not pooled and should not be compared quantitatively across studies (Figure 6).

### 3.9. Neurological and Survival Outcomes

**Figure 7:**
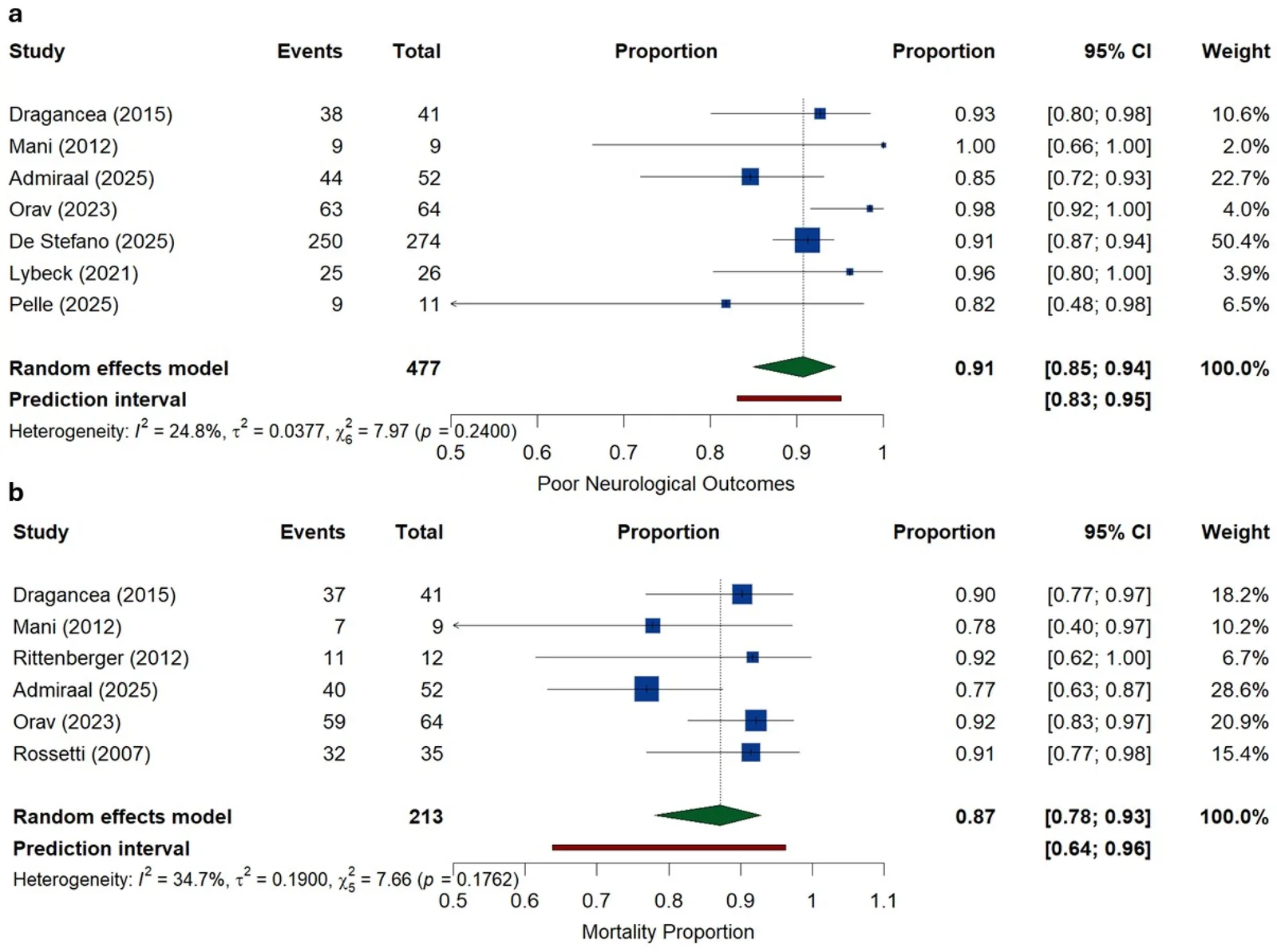
Pooled poor neurological outcome and mortality among patients with NCSE/ESE/PSE or related EEG-defined conditions. (**a**) Forest plot showing the pooled proportion of poor neurological outcome among patients with NCSE/ESE/PSE or related EEG-defined conditions after cardiac arrest [8,10,11,19,21,24,26]. Poor neurological outcome was defined according to the original study definitions, including CPC 3–5, mRS 4–6, or study-specific unfavorable outcome categories. (**b**) Forest plot showing the pooled mortality proportion among patients with NCSE/ESE/PSE or related EEG-defined conditions [8,10,19,24,28,29]. For Mani et al., the outcome analyses used the full electrographic seizure subgroup (*n* = 9) because this secondary synthesis included related EEG-defined conditions in addition to strict status epilepticus; all 9 patients had poor neurological outcome, and 7 died before hospital discharge. TELSTAR was not entered separately because its participants overlapped with the De Stefano pooled cohort.

Neurological and survival outcomes were generally poor among patients with NCSE/ESE/PSE or related EEG-defined conditions. In Dragancea et al., favorable neurological outcome at 6 months occurred in 3 of 41 patients with ESE (7.3%), while death occurred in 37 of 41 patients (90.2%) [8]. In Rittenberger et al., 11 of 12 patients with NCSE died before hospital discharge, and no patient with NCSE had a good functional outcome [29]. In Admiraal et al., 8 of 52 patients with PSE (15.4%) had mRS 0–3 at 6 months, and 12 of 52 patients (23.1%) survived to 6 months; all 12 survivors remained alive at 24 months, and seven had mRS 0–3 at 24 months [10]. The pooled proportion of poor neurological outcome across seven EEG-defined cohorts was 91% (95% CI, 85–94%), with low-to-moderate heterogeneity (I^2^ = 24.8%; τ^2^ = 0.0377; Q = 7.97; *p* = 0.240), as shown in Figure 7a. The pooled mortality proportion across six cohorts was 87% (95% CI, 78–93%), with moderate heterogeneity (I^2^ = 34.7%; τ^2^ = 0.1900; Q = 7.66; *p* = 0.176) (Figure 7b). These pooled outcome estimates combined different follow-up timepoints and functional scales and should be interpreted as reported poor-outcome and mortality estimates rather than a uniform timepoint-specific estimate. To avoid double counting, Ruijter et al. (TELSTAR) was not included separately in the pooled single-arm outcome analyses because TELSTAR participants were incorporated within the De Stefano pooled cohort; Ruijter et al. was retained only for the randomized treatment-effect analysis.

**Figure 8:**
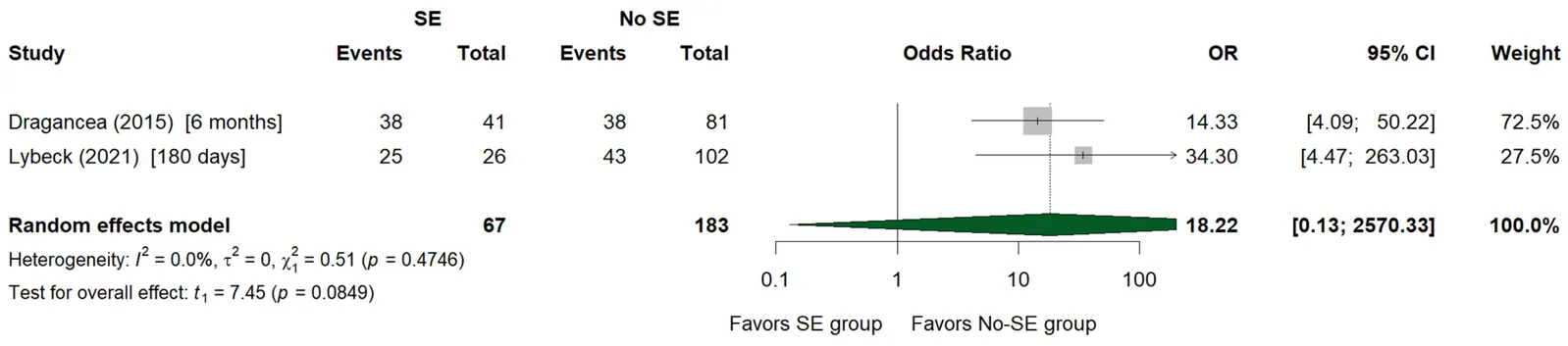
Exploratory association between SE/status and poor neurological outcome. Forest plot showing study-specific and pooled odds ratios for poor neurological outcome among patients with SE/status compared with patients without SE/status after cardiac arrest. Two studies provided comparative data [8,26].

Two studies provided comparative data for poor neurological outcomes among patients with versus without SE/status. The pooled estimate was directionally consistent with higher odds of poor neurological outcome among patients with SE/status, but it was extremely imprecise because only two studies contributed data and event distributions were sparse (OR, 18.22; 95% CI, 0.13–2570.33; I^2^ = 0.0%; *p* = 0.084). Study-specific odds ratios were 14.33 (95% CI, 4.09–50.22) in Dragancea et al. and 34.30 (95% CI, 4.47–263.03) in Lybeck et al. [8,26], as shown in Figure 8. This analysis was interpreted as exploratory and hypothesis-generating rather than confirmatory.

### 3.10. Treatment Strategies and Electrographic Response

Treatment strategies and electrographic response were heterogeneous across studies and differed by evidence type. Randomized treatment evidence was derived primarily from the TELSTAR trial [7]. Protocolized antiseizure treatment achieved ≥48 h suppression of rhythmic or periodic EEG activity in 49 of 88 patients (55.7%) in the antiseizure-treatment arm compared with 2 of 83 patients (2.4%) in the control arm. Poor neurological outcome occurred in 79 of 88 patients (89.8%) in the treatment arm and 77 of 84 patients (91.7%) in the control arm. Death occurred in 70 of 88 patients (79.5%) and 69 of 84 patients (82.1%), respectively (Table 4).

Observational treatment-response evidence was heterogeneous and was analyzed separately from randomized evidence. In Hofmeijer et al., unstandardized moderate antiseizure or sedative treatment produced temporary EEG improvement in 15 of 24 treated ESE patients (62.5%), but complete suppression did not persist for more than 6 h, and 23 of 24 treated ESE patients died [23]. In De Stefano et al., SE cessation occurred in 120 of 274 patients (43.8%) and was associated with good outcome; in the subgroup with definite SE and fewer than two poor-outcome predictors, SE cessation occurred in 32 of 52 patients (61.5%) [11]. In Orav et al. [19], EEG improvement or cessation was reported in 45 of 52 evaluable patients (86.5%) (Table 4).

### 3.11. Certainty of Evidence

Using the adapted GRADE framework, certainty of evidence was low for the primary strict NCSE/ESE/PSE prevalence estimate because of serious inconsistency and imprecision. Certainty was very low for sensitivity analyses including selected/historical cohorts, broad electrographic seizure/status prevalence, broad epileptiform/IIC prevalence, comparative SE/status outcome association, and observational treatment-response evidence. Certainty was low for pooled poor neurological outcome and mortality because of observational designs, withdrawal-of-life-sustaining-therapy confounding, and variable outcome timepoints. Certainty was moderate for the TELSTAR randomized comparison of protocolized suppression versus standard care, downgraded for open-label treatment despite blinded outcome assessment (Table 5).

## 4. Discussion

NCSE/ESE/PSE was 22% among EEG-monitored comatose adult survivors of cardiac arrest, but substantial heterogeneity and the wide prediction interval indicate considerable uncertainty across clinical settings, related to EEG timing, monitoring duration, denominator selection, and diagnostic criteria. Poor neurological outcome and mortality were frequent; nevertheless, favorable outcomes were reported in selected patients with preserved or recovered EEG background, later SE onset, fewer multimodal poor-outcome markers, and electrographic cessation.

The prevalence estimate from the present meta-analysis is consistent with the broad range reported in the previous post-cardiac arrest EEG literature but provides a focused estimate for strict NCSE/ESE/PSE constructs. Earlier studies and reviews reported electrographic seizures or status epilepticus in approximately 9–35% of comatose post-cardiac arrest patients, while a focused review of NCSE after ROSC identified 11 cohort studies including 1092 patients and reported NCSE frequencies of approximately 1–20% [7,9,23]. In the present analysis, the primary strict prevalence estimate was 22%, and the expanded sensitivity analysis, which included selected, historical, or restricted cohorts, yielded an identical pooled estimate of 22% (95% CI, 16–31%). This stability supports the overall direction of the finding, although the prediction intervals and study-level heterogeneity suggest that local estimates may be strongly modified by EEG acquisition and diagnostic thresholds.

The observed heterogeneity was clinically and methodologically expected. Studies differed in EEG modality, timing, and monitoring duration, ranging from early cEEG within approximately 8–15 h to later intermittent EEG after temperature management or rewarming. Because post-anoxic EEG patterns evolve during the first 72 h after ROSC, short recordings may miss intermittent seizures, whereas prolonged cEEG may detect rhythmic/periodic patterns that do not meet strict electrographic status criteria [6,20,21,24,25,29].

Terminological variation further contributed to heterogeneity. ACNS terminology separates electrographic seizures, ESE, electroclinical seizures/status epilepticus, brief potentially ictal rhythmic discharges, and IIC patterns, whereas older post-cardiac arrest studies often grouped generalized periodic discharges, rhythmic delta activity, or epileptiform burst-suppression under SE [6,20,23,28]. The Salzburg criteria provide a structured NCSE framework, but post-anoxic rhythmic or periodic discharges may reflect an ictal process, severe hypoxic–ischemic injury, or both [4,5]. Therefore, the conditional phenotype distributions reported by Backman et al., De Stefano et al., and Orav et al. were clinically informative but not epidemiologically interchangeable [11,19,20].

The broad electrographic seizure/status and broad epileptiform/IIC analyses should therefore be interpreted as descriptive rather than competing prevalence estimates. Their pooled estimates reflected different numerator definitions, including isolated seizures, combined seizure/status constructs, generalized or lateralized periodic discharges, rhythmic delta activity, and clinically abstracted epileptiform/IIC patterns. These findings identify high-risk neurophysiological states, but their biological and therapeutic equivalence to strict NCSE/ESE/PSE remains unproven [7,22,31].

The pooled poor-outcome estimate of 91% and mortality estimate of 87% confirm that post-anoxic NCSE/ESE/PSE usually occurs in a population with severe hypoxic–ischemic brain injury. These findings align with prior reports showing high case fatality in postanoxic ESE and with post-resuscitation guidelines that consider unequivocal electrographic seizures, highly malignant EEG backgrounds, absent reactivity, suppression, and burst-suppression as adverse prognostic features when interpreted in the appropriate temporal and clinical context [2,3,8,28,32,33,34]. In the Target Temperature Management trial EEG cohort, standardized interpretation showed that highly malignant EEG patterns after rewarming were strongly associated with poor outcome, and subsequent analyses showed that highly malignant routine EEG had high specificity for poor prognosis but limited sensitivity [33,34]. These findings are concordant with our finding that SE after cardiac arrest is usually embedded within a broader pattern of severe brain injury rather than acting as an isolated prognostic marker.

However, the present findings also support caution against using NCSE/ESE/PSE as a single determinant of futility. In Dragancea et al., all patients with a discontinuous background before ESE died, whereas survival with CPC 1–2 occurred in a small subgroup with continuous background before ESE [8]. In the TTM2 cEEG substudy, Admiraal et al. reported that 8 of 20 patients with a favorable PSE profile achieved good functional outcome at 6 months, whereas all patients without this profile had poor outcome [10]. In the De Stefano pooled individual-patient analysis, good recovery occurred in only 8.8% of the overall definite/possible SE cohort, but increased to 25% after exclusion of patients with two or more poor-outcome ERC/ESICM criteria [11]. These data suggest that EEG background continuity, timing of SE onset, absence of multiple poor prognostic markers, and electrographic seizure cessation may refine prognosis beyond the presence or absence of SE alone.

Current guidelines emphasize delayed, multimodal prognostication, avoidance of sedation and temperature-related confounding, and integration of clinical examination, EEG, somatosensory evoked potentials, biomarkers, and neuroimaging rather than reliance on a single predictor [35,36]. The self-fulfilling prophecy remains a major concern in this field because perceived poor prognosis may influence withdrawal of life-sustaining therapy, which then determines mortality [35,37]. This concern is particularly relevant to NCSE/ESE/PSE studies, where treatment intensity, prognostic interpretation, and withdrawal decisions may be directly influenced by EEG findings.

Treatment evidence indicates a dissociation between electrographic suppression and clinical recovery at the population level, while leaving open the possibility of benefit in selected patients. TELSTAR tested a broad rhythmic/periodic EEG-pattern strategy rather than a narrowly defined electrographic SE phenotype; ILCOR subsequently noted that many treated patterns were low-frequency generalized periodic discharges without evolution and may have represented severe hypoxic–ischemic injury rather than a modifiable ictal process [7,38]. Accordingly, current resuscitation guidance does not support prophylactic antiseizure therapy after cardiac arrest, but suggests treatment of clinically apparent or electrographic seizures and gives only weak, low-certainty support for treating IIC patterns [38]. Observational data suggest that responsiveness may depend on preserved cortical background, with levetiracetam and valproate associated with resolution of epileptiform activity only in continuous-background states [39]. Overall, the evidence supports phenotype-guided escalation rather than uniform aggressive suppression of all rhythmic or periodic patterns [40].

The distinction between treating “seizures” and treating “injury-pattern EEG” is central. Rhythmic or periodic patterns may lie on a continuum from interictal injury markers to potentially ictal activity, and the likelihood of treatment benefit may depend on discharge frequency, evolution, background continuity, reactivity, timing, and burden. ACNS terminology and recent consensus approaches support this separation, but the evidence base remains insufficient to define an actionable treatment threshold for post-anoxic IIC patterns [6,11,31]. Future trials should therefore avoid enrolling all rhythmic or periodic patterns as a single entity and should stratify by ACNS-defined definite SE, possible SE/IIC, EEG background, timing, and multimodal prognostic profile.

The included biomarker and prognostic EEG studies suggest that NCSE/ESE/PSE should be interpreted as part of a broader post-cardiac arrest brain injury phenotype. Lybeck et al. found that ESE was associated with higher serum neurofilament light chain at 72 h after cardiac arrest, suggesting an association between ESE and neuronal injury, although residual confounding by initial injury severity could not be excluded [26]. Pelle et al. reported that neuron-specific enolase thresholds for poor outcome varied according to EEG pattern, reinforcing that biomarker interpretation may differ across EEG-defined injury states [21]. Peluso et al. showed that delayed epileptic EEG deterioration occurred in a selected subgroup without early malignant or epileptic EEG features and was associated with worse outcomes, suggesting that EEG evolution over time may provide additional prognostic information beyond the first recording [18].

These observations align with the broader post-cardiac arrest literature. Post-cardiac arrest brain injury is a dynamic process involving ischemia–reperfusion injury, excitotoxicity, mitochondrial dysfunction, inflammation, cerebral edema, and delayed neuronal injury [32]. EEG provides a real-time measure of cortical function and may capture both injury severity and potentially modifiable epileptiform activity [41,42]. Therefore, a binary interpretation of NCSE/ESE/PSE as either “treatable seizure” or “irreversible injury marker” is probably insufficient. A more plausible model is that electrographic status epilepticus reflects a heterogeneous state in which some patients have irreversible cortical injury, while others retain enough background organization and multimodal prognostic reserve to justify continued treatment and observation.

### 4.1. Clinical Implications

The first clinical implication is that EEG monitoring should be considered an important diagnostic component rather than an optional prognostic adjunct in comatose post-cardiac arrest patients. A pooled strict NCSE/ESE/PSE prevalence of 22% indicates that a clinically important proportion of patients may have electrographic seizures or status epilepticus that cannot be reliably detected by clinical examination, particularly during sedation, neuromuscular blockade, or temperature control [2,3,41]. Where cEEG is not available, repeated routine EEG may still provide clinically useful information, but short recordings may miss intermittent seizures and may inadequately characterize burden, evolution, or response to treatment [25,41].

The second implication is that NCSE/ESE/PSE should not be used in isolation to justify withdrawal of life-sustaining therapy. Although the pooled prognosis was poor, recovery was observed in selected patients, particularly those with continuous EEG background before SE onset, later SE onset, preserved multimodal prognostic markers, or electrographic cessation [8,10,11,40]. Clinical decision-making should therefore integrate EEG phenotype, timing, background continuity/reactivity, somatosensory evoked potentials, biomarkers, neuroimaging, clinical examination, and sedation clearance. This is consistent with guideline-supported multimodal prognostication and mitigates the risk of circular reasoning in which an EEG abnormality simultaneously informs withdrawal decisions and becomes a predictor of death [35,36,37].

The third implication is that antiseizure treatment should be individualized rather than reflexively escalated in all patients with rhythmic or periodic EEG patterns. TELSTAR does not support routine aggressive suppression of all rhythmic/periodic patterns after cardiac arrest; however, observational data support further evaluation of treatment in patients with definite SE, continuous or recovered EEG background, fewer poor-outcome markers, and potential for electrographic cessation [10,11,40]. In practice, this supports a phenotype-guided approach: aggressive treatment may be considered in patients with favorable multimodal features, whereas prolonged anesthetic coma may be less justifiable in patients with suppressed/burst-suppression background, absent brainstem/SSEP responses, high injury biomarkers, or multiple convergent poor-outcome markers.

### 4.2. Future Directions

Future studies should standardize four methodological domains. First, EEG-defined exposures should be reported using ACNS 2021 terminology, with explicit separation of electrographic seizures, ESE, electroclinical SE, possible SE/IIC, generalized periodic discharges, lateralized periodic discharges, rhythmic delta activity, and burst-suppression phenotypes [6]. Second, denominator reporting should distinguish all cardiac arrest patients, all comatose survivors, all EEG-monitored patients, and selected SE/RPP cohorts. This distinction was essential in the present review because selected cohorts and unselected EEG-monitored cohorts answer different clinical questions.

Third, future treatment trials should use enrichment rather than unselected RPP inclusion. Candidate enrichment criteria include definite ACNS SE, continuous or recovered EEG background before SE onset, later SE onset, preserved N20 responses, absence of multiple ERC/ESICM poor-outcome markers, and an early electrographic response to first- or second-line therapy [10,11,40]. Treatment endpoints should include not only suppression of rhythmic/periodic activity but also durable SE cessation, recurrence, sedative exposure, mechanical ventilation duration, ICU complications, delayed awakening, and functional outcomes at 6 months or later. Fourth, withdrawal-of-life-sustaining-therapy policies should be protocolized and reported in detail, ideally with blinded outcome adjudication and prespecified separation between prognostic EEG interpretation and treatment decisions [35,36,37].

Individual-patient-data meta-analysis may be particularly valuable in this field. Study-level meta-analysis cannot adequately evaluate interactions between EEG background, SE timing, sedative exposure, biomarker trajectory, SSEP findings, treatment intensity, and withdrawal decisions. Quantitative EEG and machine-learning approaches may also refine risk stratification, but these models should be developed with transparent definitions, external validation, and safeguards against incorporation of self-fulfilling withdrawal patterns into prediction targets [42,43].

### 4.3. Strengths and Limitations

This review has several strengths. The review separated strict NCSE/ESE/PSE from broader electrographic seizure/status and epileptiform/IIC constructs, reducing the risk of pooling biologically and diagnostically different EEG entities. Cohort overlap was explicitly addressed, with Dragancea and Backman, Admiraal Neurology and Admiraal Resuscitation, and Ruijter and De Stefano handled according to endpoint-specific analytical roles. The review also separated randomized treatment evidence from observational treatment-response evidence, which was necessary because observational studies were highly vulnerable to confounding by indication and withdrawal-of-life-sustaining-therapy practices.

Several limitations should be considered. The evidence base was heterogeneous with respect to EEG modality, EEG timing, monitoring duration, diagnostic criteria, sedation exposure, temperature-management era, and outcome timepoint. Several studies also used selected denominators, including SE-selected cohorts, EEG-selected cohorts, biomarker subsets, or trial-entry rhythmic/periodic pattern cohorts, limiting comparability with unselected EEG-monitored post-cardiac arrest populations. Some meta-analyses included few studies, particularly the comparative SE/status analysis, and neurological outcome definitions varied across CPC, modified Rankin Scale, hospital discharge, 3-month, 6-month, and longer-term follow-up. The pooled poor-outcome and mortality estimates may also be affected by self-fulfilling prophecy because EEG abnormalities may have contributed to withdrawal decisions in several observational cohorts. Observational treatment-response studies could not support causal inference because treatment intensity was confounded by EEG severity, baseline injury, and coexisting poor prognostic markers. Publication bias and small-study effects were difficult to assess because most analyses included fewer than 10 studies and heterogeneity reflected real differences in monitoring and diagnostic strategy.

## 5. Conclusions

NCSE, ESE, and PSE were identified in a pooled 22% of EEG-monitored comatose survivors of cardiac arrest, although substantial between-study heterogeneity limits the precision and generalizability of this estimate. These EEG-defined conditions were associated with high rates of poor neurological outcome and mortality, but prognosis was not uniformly futile. Favorable outcome appeared concentrated in selected patients with favorable EEG background, later SE onset, fewer multimodal poor-outcome markers, and electrographic cessation. The available randomized evidence did not support routine aggressive suppression of all rhythmic or periodic EEG patterns, whereas observational and pooled patient-level data suggest that treatment responsiveness may vary by electroclinical phenotype. Future research should use standardized ACNS-based definitions, consistent denominators, protocolized multimodal prognostication, transparent withdrawal-of-life-sustaining-therapy policies, and enriched randomized designs to determine whether specific electroclinical phenotypes are associated with treatment-responsive outcomes.

## Figures and Tables

**Table 1:** Characteristics of included studies.

Study	Country/Region	Centers/Setting	Design	Recruitment Period	Population	Analysis Sample	Follow-Up	Main Findings
Pelle et al., 2025 [21]	France	Single tertiary CA center	Retrospective registry cohort	February 2017–December 2022	Adult patients comatose 72 h after CA	*N* = 155; all had EEG classification by Westhall categories	3-month CPC	Electrographic seizure/status occurred in 11/155; poor 3-month CPC was more frequent in patients with malignant/highly malignant EEG patterns; NSE thresholds varied by EEG pattern.
Backman et al., 2017 [20]	Sweden	Skåne University Hospital, Lund	Retrospective cohort analysis	January 2008–March 2013	Adult CA patients treated with TTM and simplified cEEG who developed ESE	*N* = 41 with ESE from a 127-patient monitored CA cohort	6-month CPC	ESE occurred early and frequently; 3/4 survivors had unequivocal ESE from a continuous background; unequivocal and possible ESE did not differ materially in survival.
Peluso et al., 2024 [18]	Belgium	Erasme Hospital, Brussels	Retrospective monocentric cohort	January 2012–December 2020	Adult CA ICU patients with initially non-malignant, non-epileptic EEG within 24 h	*N* = 188 selected from 527 EEG-monitored CA patients	ICU mortality; 3-month neurological outcome	Delayed epileptic deterioration occurred in 30/188 and was associated with higher ICU mortality and unfavorable outcome; all patients with delayed background deterioration died in ICU.
Orav et al., 2023 [19]	Austria	Christian Doppler University Hospital, Salzburg	Prospective SE database cohort with retrospective EEG review	February 2019–March 2023	Patients with possible or definitive SE from hypoxic brain injury after CA	*N* = 64 post-hypoxic SE patients	1-month CPC/mortality	Mortality was 92%; only one patient had good CPC; outcomes did not differ significantly among electrographic SE, electroclinical SE, and IIC groups.
Dragancea et al., 2015 [8]	Sweden	Skåne University Hospital, Lund	Retrospective single-center cohort	January 2008–February 2013	Consecutive CA patients treated with TTM and simplified cEEG	*N* = 127; ESE = 41	6-month CPC	ESE occurred in 32%; all patients with discontinuous background before ESE died, whereas 4 patients with continuous background survived, 3 with CPC 1–2.
Faro et al., 2019 [22]	United States	Two Pittsburgh academic centers	Retrospective cohort	January 2011–October 2014	Comatose post-arrest ICU patients with different EEG monitoring strategies	*N* = 818; any epileptiform pattern = 258	Hospital discharge	Any epileptiform pattern was associated with poor outcomes; only 2/258 with epileptiform activity were discharged with mRS 0–2.
Hofmeijer et al., 2014 [23]	Netherlands	Two hospitals	Retrospective treatment analysis within prospective cEEG cohort	1 June 2010–31 March 2013	Comatose CA patients undergoing continuous EEG; treated and untreated ESE subgroups	*N* = 139; treated with AED = 31; treated ESE = 24	6-month CPC	Unstandardized moderate treatment temporarily improved EEG patterns but was not associated with improved outcomes; 23/24 treated ESE patients died.
Mani et al., 2012 [24]	United States	University of Pennsylvania	Single-center retrospective cEEG cohort	May 2005–January 2009	Comatose adult PCAS patients treated with TH and cEEG	*N* = 38	Hospital discharge CPC/mortality	Electrographic seizures occurred in 9/38 and SE in 7/38; all patients with electrographic seizures had poor neurological outcome at discharge, and 7/9 died in hospital.
Admiraal et al., 2025 [10]	Multinational Europe	Seven TTM2 cEEG sites	Prospective observational substudy	2017–2020	Adult comatose OHCA patients in TTM2 substudy with cEEG monitoring	*N* = 191; PSE = 52	6 and 24 months; primary 6-month mRS	PSE occurred in 52/191; favorable PSE profile was present in 20/52 and 8/20 had good 6-month outcome; all without favorable profile had poor outcome.
Ruijter et al., 2022 [7]	Netherlands/Belgium	11 ICUs	Open-label randomized controlled trial with blinded outcome assessment	NR	Comatose CA survivors with rhythmic/periodic EEG patterns on cEEG	*N* = 172; antiseizure treatment = 88; control = 84	3-month CPC; mortality; ICU LOS; ventilation duration	Protocolized suppression increased EEG suppression but did not reduce poor 3-month neurological outcome or mortality.
Urbano et al., 2022 [25]	Switzerland	CERTA trial subgroup	Randomized EEG monitoring strategy analysis	April 2018–September 2019	Comatose adults after CA undergoing cEEG or two routine EEG recordings	*N* = 112; cEEG = 60; rEEG = 52	6-month mortality and CPC	Mortality and good CPC outcome were comparable between cEEG and repeated rEEG after adjustment.
Lybeck et al., 2021 [26]	Sweden	TTM trial cEEG/biomarker cohort	Post hoc cohort analysis	2010–2013	Comatose CA patients with cEEG and serum NfL/GFAP biomarkers	*N* = 128; ESE = 26	180-day CPC; biomarkers at 24/48/72 h	ESE was independently associated with higher NfL at 72 h and with poor CPC; associations with GFAP were less clear.
Admiraal et al., 2025 [27]	Multinational Europe	TTM2 cEEG substudy sites	Observational prognostic substudy	2017–2020	Consecutive comatose resuscitated patients in TTM2 cEEG substudy	*N* = 191	6-month mRS	Combining early and late EEG predictors increased sensitivity for poor outcome while retaining 100% specificity in this cohort.
Rossetti et al., 2007 [28]	Switzerland	University ICU, Lausanne	Retrospective observational cohort	June 1999–June 2006	Comatose survivors of CA aged >16 years with EEG in early hospital days	*N* = 107 with EEG from 166 postanoxic patients; EEG-selected cohort	Hospital discharge survival/CPC	PSE was associated with death independently of rhythm and hypothermia; mortality was 32/35 with PSE.
Rittenberger et al., 2012 [29]	United States	Pittsburgh	Consecutive cohort/quality-improvement database	August 2009–November 2010	Comatose post-CA subjects treated with TH and cEEG	*N* = 101	Hospital discharge CPC/mRS; survival; command-following	NCSE occurred in 12/101, usually early after cEEG initiation; only one NCSE patient survived and none had good functional outcome.
Shih et al., 2025 [30]	United States	Urban academic safety-net hospital	Retrospective cohort	July 2016–April 2022	Comatose OHCA patients; substance-use-related vs. non-substance arrests	*N* = 253; SURCA = 99; EEG-monitored = 166	Hospital discharge CPC/mortality	SURCA patients had higher electrographic seizure and GPD rates on EEG but similar poor neurological outcome and mortality compared with non-SURCA patients.
De Stefano et al., 2025 [11]	Multinational Europe	TELSTAR plus Brussels/Geneva registries	Pooled individual-patient data analysis	NR	Comatose CA patients with ACNS definite or possible SE within 72 h	302 screened across TELSTAR, Brussels, and Geneva; 274 met ACNS definite/possible SE criteria; <2 poor-outcome predictors subgroup *N* = 94	3-month CPC	Overall good recovery was 8.8%; after excluding patients with ≥2 poor-outcome criteria, good outcome reached 25%; SE cessation remained associated with good outcome.

ACNS, American Clinical Neurophysiology Society; AED, antiepileptic drug; CA, cardiac arrest; cEEG, continuous electroencephalography; CERTA, Continuous EEG Randomized Trial in Adults; CPC, Cerebral Performance Category; EEG, electroencephalography; ESE, electrographic status epilepticus; GFAP, glial fibrillary acidic protein; GPD, generalized periodic discharge; ICU, intensive care unit; IIC, ictal–interictal continuum; LOS, length of stay; mRS, modified Rankin Scale; NCSE, nonconvulsive status epilepticus; NfL, neurofilament light chain; NR, not reported; NSE, neuron-specific enolase; OHCA, out-of-hospital cardiac arrest; PCAS, post-cardiac arrest syndrome; PSE, post-anoxic status epilepticus; rEEG, routine electroencephalography; SE, status epilepticus; SURCA, substance-use-related cardiac arrest; TELSTAR, Treatment of Electroencephalographic Status Epilepticus After Cardiopulmonary Resuscitation; TH, therapeutic hypothermia; TTM, targeted temperature management.

**Table 2:** Baseline demographic and cardiac arrest characteristics.

Study	Analysis Cohort/Group	*N*	Age	Male, *n* (%)	OHCA, *n* (%)	Shockable Rhythm, *n* (%)	Witnessed Arrest, *n* (%)	Time to ROSC, min	TTM Use/Target
Pelle et al., 2025 [21]	Overall cohort	155	64 [53–72.5]	115 (74.2%)	128 (82.6%)	75 (48.4%)	NR	NR	TTM at 33 °C from 2017–2021 or 36 °C from 2021–2022 for 24 h in patients remaining comatose after ROSC
	Good CPC 1–2 at 3 months	39	58 [50–65.5]	30 (76.9%)	32 (82.1%)	28 (71.8%)	NR	18 [11–24]	
	Poor CPC 3–5 at 3 months	116	66.5 [54.8–75]	85 (73.3%)	96 (82.8%)	47 (40.5%)	NR	25 [21.8–34]	
Dragancea et al., 2015 [8]	Overall EEG-monitored cohort	127	NR	94 (74.0%)	108 (85.0%)	81 (63.8%)	NR	NR	All included patients received TTM; target strata: 33 °C 111/127; 36 °C 16/127
	ESE	41	68 [60–72]	28 (68.3%)	37 (90.2%)	26 (65.0%)	NR	28 [21–40]	All ESE patients received TTM; target strata: 33 °C 38/41; 36 °C 3/41
	No ESE	86	65 [56–73]	66 (76.7%)	71 (82.6%)	55 (64.0%)	NR	20 [13–32]	All no-ESE patients received TTM; target strata: 33 °C 73/86; 36 °C 13/86
Backman et al., 2017 [20]	All ESE	41	68 [60–72]	28 (68.3%)	NR	26 (65.0%)	NR	27 [20–40]	All included ESE patients received TTM; target strata: 33 °C 38/41; 36 °C 3/41
	Unequivocal ESE	22	67 [58–70]	15 (68.2%)	NR	13 (61.9%)	NR	25 [19–36]	All received TTM; 33 °C 22/22
	Possible ESE	19	69 [61–74]	13 (68.4%)	NR	13 (68.4%)	NR	32 [25–42]	All received TTM; target strata: 33 °C 16/19; 36 °C 3/19
Peluso et al., 2024 [18]	Selected non-early-malignant EEG cohort	188	65 [55–75]	132 (70.2%)	124 (66.0%)	100 (53.2%)	NR	18 [10–30]	TTM at 33 °C for 24 h unless contraindicated; passive rewarming 0.3–0.5 °C/h; sedation discontinued at 37 °C
	Delayed epileptic deterioration	30	67 [55–77]	24 (80.0%)	27 (90.0%)	18 (60.0%)	NR	25 [18–33]	
Orav et al., 2023 [19]	Post-hypoxic SE cohort	64	66 [62–74]	42 (65.6%)	52 (81.2%)	28 (43.8%)	NR	20 [15–32]	TTM 32–36 °C for 24 h followed by controlled rewarming in comatose patients
Faro et al., 2019 [22]	Overall cohort	818	61 [50–72]	491 (60.0%)	528 (64.5%)	218 (26.7%)	NR	NR	Center 1 used TTM 33 °C for 24 h followed by slow rewarming at 0.25 °C/h; Center 2 protocol not equivalently detailed.
Hofmeijer et al., 2014 [23]	Overall cEEG cohort	139	NR	NR	124 (89.2%)	97 (69.8%)	NR	NR	All included patients treated with mild therapeutic hypothermia maintained for 24 h; sedation discontinued after normothermia when possible.
	AED-treated	31	64 ± 11	NR	29 (93.5%)	21 (67.7%)	NR	NR	
	No AED treatment	108	65 ± 12	NR	95 (88.0%)	76 (70.4%)	NR	NR	
Mani et al., 2012 [24]	Overall cEEG cohort	38	58 [45–65]	20 (52.6%)	NR	14 (43.8%)	NR	20 [10–30]	Therapeutic hypothermia target 33 °C for 24 h, followed by active rewarming to 36 °C over 12–18 h
Admiraal et al., 2025 [10]	TTM2 cEEG cohort	191	68 [61–75]	144 (75.4%)	191 (100.0%)	130 (68.1%)	179 (93.7%)	NR	TTM2 randomization to 33 °C hypothermia or normothermia with early fever control; 33 °C 94/191
	PSE cohort	52	70 [63–77]	34 (65.4%)	NR	NR	NR	NR	
Ruijter et al., 2022 [7]	Antiseizure-treatment arm	88	64 [56–73]	60 (68.2%)	84 (95.5%)	51 (60.0%)	62 (70.5%)	16 [10–30]	TTM: 33 °C 21/88; 33–36 °C 67/88
	Control arm	84	66 [59–74]	58 (69.9%)	78 (94.0%)	58 (71.6%)	53 (63.9%)	19 [12–25]	TTM: 33 °C 20/83; 33–36 °C 63/83
Urbano et al., 2022 [25]	cEEG arm	60	64.3 ± 12.1	46 (76.7%)	NR	31 (51.7%)	NR	27 [13–45]	TTM 36 °C for 24 h in all post-CA patients
	Repeated routine EEG arm	52	64.2 ± 14.8	35 (67.3%)	NR	19 (36.5%)	NR	18 [10–26]	TTM 36 °C for 24 h in all post-CA patients
Lybeck et al., 2021 [26]	ESE	26	72 [65–81]	21(80.8%)	NR	15 (57.7%)	24 (92.3%)	34 [23–46]	TTM trial care; all included; target 33 °C 14/26
	No ESE	102	64 [56–71]	82 (80.4%)	NR	83 (81.4%)	95 (93.1%)	25 [16–35]	TTM trial care; all included; target 33 °C 50/102
Rossetti et al., 2007 [28]	EEG cohort	107	NR	81 (75.7%)	NR	69 (64.5%)	NR	NR	Therapeutic hypothermia to 33 °C for 24 h used in 63/107 EEG patients; older mixed pre-/TTM era
Rittenberger et al., 2012 [29]	Overall cohort	101	57 ± 15	55 (54.5%)	78 (77.2%)	39 (38.6%)	NR	NR	All received TH; target 33 °C for 24 h; rewarming 0.2–0.3 °C/h
	NCSE	12	55 ± 15	4 (33.3%)	10 (83.3%)	4 (33.3%)	NR	NR	
Shih et al., 2025 [30]	Overall OHCA cohort	253	59 ± 18	183 (72.3%)	253 (100.0%)	52 (21.0%)	165 (65.7%)	20 [23]	Targeted temperature control per institutional protocol
	SURCA	99	49 ± 12	81 (81.8%)	99 (100.0%)	11 (11.2%)	55 (55.6%)	20 [23]	
	Non-SURCA	154	66 ± 18	104 (67.5%)	154 (100.0%)	41 (27.3%)	110 (71.4%)	22 [24]	
Admiraal et al., 2025 [27]	TTM2 cEEG cohort	191	68 [61–75]	144 (75.4%)	191 (100.0%)	130 (68.1%)	179 (93.7%)	NR	TTM2 randomization to 33 °C hypothermia or normothermia with early fever control; 33 °C 94/191
De Stefano et al., 2025 [11]	Whole definite/possible SE cohort	274	66 [55–75]	190 (69.3%)	244 (89.1%)	143 (52.2%)	NR	20 [15–30]	TTM to 33–34 °C *n* = 98
	<2 ERC/ESICM poor-prognosis criteria	94	63 [56–74]	62 (66.0%)	84 (89.4%)	66 (70.2%)	NR	17 [15–25]	TTM to 33–34 °C *n* = 24

AED, antiepileptic drug; CA, cardiac arrest; cEEG, continuous electroencephalography; CPC, Cerebral Performance Category; EEG, electroencephalography; ERC/ESICM, European Resuscitation Council/European Society of Intensive Care Medicine; ESE, electrographic status epilepticus; NCSE, nonconvulsive status epilepticus; NR, not reported; OHCA, out-of-hospital cardiac arrest; PSE, post-anoxic status epilepticus; ROSC, return of spontaneous circulation; SE, status epilepticus; SURCA, substance-use-related cardiac arrest; TH, therapeutic hypothermia; TTM, targeted temperature management; TTM2, Targeted Temperature Management 2 trial.

**Table 3:** EEG monitoring methods and diagnostic definitions.

Study	EEG Modality	EEG Start	EEG Duration	Diagnostic Framework	NCSE/ESE/PSE Definition	IIC/RPP Definition	ACNS Used	EEG Review/Blinding
Pelle et al., 2025 [21]	Video-EEG recorded in ICU using Deltamed Coherence or Micromed Brain Quick devices	First EEG median 2 [1–3] days after CA, after end of TTM	Minimum 20 min	Westhall classification; ACNS terminology used for seizure/status and EEG descriptors	Electrographic seizures/status reported but strict definition not extracted	RPPs reported	Yes	Two neurophysiologists re-analyzed EEG recordings blinded to other prognostic markers and outcome
Backman et al., 2017 [20]	Simplified 2-channel cEEG: F3–P3/F4–P4 or C3–P3/C4–P4; filters 1–70 Hz	8 [6–11] h from CA to cEEG initiation	117 [75–173] h	ACNS terminology used for retrospective characterization	Unequivocal ESE: bilateral spike/sharp waves ≥ 3 Hz for ≥50% of 30 min or evolving discharges > 4 Hz for ≥50% of 30 min; possible ESE: rhythmic spike/sharp waves or periodic discharges ≥ 1 Hz for ≥30 min	Possible ESE/IIC *n* = 19	Yes	A senior consultant in clinical neurophysiology retrospectively reviewed cEEG recordings blinded to outcome and other clinical parameters
Dragancea et al., 2015 [8]	Simplified 2-channel bipolar cEEG: C3–P3/C4–P4 or F3–P3/F4–P4, displaying original EEG and amplitude-integrated EEG trends	cEEG from arrival at ICU	NR	Strict retrospective ESE criteria	Continuous rhythmic polyspike/spike/sharp-wave or periodic discharges with typical frequency ≥ 1 Hz for 30 min, or unequivocal seizures recurring for 30 min, according to ACNS terminology	NR	Yes	Neurophysiologist blinded to outcome reanalyzed cEEG
Peluso et al., 2024 [18]	Continuous EEG; 21 electrodes according to international 10–20 system; BrainRT software	Started as soon as possible after admission and continued at least until end of rewarming; selected cohort required non-malignant early EEG within 24 h	46 [37–55] h overall	ACNS standardized critical care EEG terminology; study-specific delayed deterioration definitions	Delayed epileptic deterioration: new sporadic ED, periodic discharges, or electrographic seizures/status epilepticus ≥ 24 h after CA	GPD/LPD/BIPD and sporadic ED reported	Yes	Single neurophysiologist review; blinding not reported
Orav et al., 2023 [19]	Intermittent EEG using 21 electrodes according to the 10–20 system; repeated EEGs if clinically indicated	First EEG median 46 [35.5–63.5] h after CA; possible/definitive SE diagnosis median 47 [39–72] h	Minimum 20 min	ACNS and Salzburg criteria; ILAE semiology classification for definitive SE	Definitive electrographic SE, definitive electroclinical SE, possible electrographic SE/IIC, or no SE according to ACNS and Salzburg criteria	IIC *n* = 15/64	Yes	Two independent raters blinded to outcome; consensus with third reviewer if disagreement
Faro et al., 2019 [22]	Routine cEEG at one center; clinician-driven spot EEG at another	Center 1 routine cEEG; Center 2 clinician-driven spot EEG if seizure suspected	Center 1 median cEEG 2 [0–3] days; Center 2 median spot EEG exposure 0 [0–1] days; spot EEG generally at most once daily	Study-specific epileptiform pattern classification	Seizures as subtype of ictal–interictal spectrum	Any epileptiform/IIC pattern *n* = 258	NR	EEG data were reviewed and classified; outcome blinding not reported
Hofmeijer et al., 2014 [23]	Continuous EEG using 19 channels according to the international 10–20 system	Started within 12 h after cardiac arrest; 24 h epochs analyzed and treatment-period EEG reviewed	Continued until consciousness recovery, death, or up to 5 days	Study criteria for ESE: unequivocal seizures or GPD > 30 min	ESE defined as unequivocal seizures, generalized spike-wave ≥ 3/s, clearly evolving discharges ≥ 4/s, or rhythmic/periodic patterns including generalized/lateralized periodic discharges or rhythmic delta activity for >30 min	GPD and burst suppression with epileptiform bursts included	NR	24 h epochs independently analyzed by two investigators blinded to clinical condition, epoch timing, and outcome; treatment-period EEGs reviewed by two observers blinded to outcome
Mani et al., 2012 [24]	cEEG or frequent routine EEG; international 10–20 electrode configuration with 16–18 EEG channels	14.8 [7.5–21.3] h after arrest	47.5 [38–67.6] h	Study-specific cEEG terminology	Status epilepticus defined as seizure activity > 30 min	Interictal epileptiform activity, PLEDs, GPEDs	NR	Raw EEG waveforms cleared of annotations/clinical information and reviewed independently by two fellowship-trained electroencephalographers blinded to clinical course and neurological outcomes
Admiraal et al., 2025 [10]	Continuous EEG with at least 6 electrodes according to 10–20 system plus reference and ground	cEEG monitoring started median 8 [6–9] h after CA	Central review in consecutive 6 h epochs from 6 ± 3 h to 72 ± 3 h after CA	ACNS standardized terminology	PSE present if patterns occurred continuously ≥10 min or ≥20% of a 60 min period; definite PSE: rhythmic/periodic epileptiform or sharply contoured discharges > 2.5 Hz or definite evolution; possible PSE: periodic/spike-wave > 1 Hz or ≥0.5 Hz with plus/fluctuation, or LRDA > 1 Hz with plus/fluctuation	Possible PSE included in profile	Yes, ACNS 2021	Certified EEG specialist blinded to outcome and clinical data
Ruijter et al., 2022 [7]	Continuous EEG	Started < 24 h after ROSC; RPP detected median 35 h after CA	cEEG started < 24 h after ROSC and continued for at least 3 days, until ICU discharge, or until RPP activity was extinguished	Trial RPP definition: PD, RDA, spike/sharp-wave ≥ 0.5 Hz	Not limited to strict SE; rhythmic/periodic patterns targeted	RPPs included periodic discharges, rhythmic delta activity, spike-and-wave, or sharp-and-wave patterns ≥ 0.5 Hz; inclusion required continuous activity ≥ 30 min or intermittent activity ≥ 5 min recurring at least twice at intervals < 60 min	NR	Endpoint blinded; EEG patterns treated open-label
Urbano et al., 2022 [25]	cEEG 30–48 h vs. two rEEG recordings 20–30 min	Time to EEG after admission: cEEG median 22.0 h vs. rEEG 24.6 h (not ROSC timing)	cEEG 30–48 h vs. two rEEGs 20–30 min each within same timeframe	ACNS recommendations at trial time	Seizure/SE detected	IIC *n* = 18/112	NR	Outcome assessed blinded
Lybeck et al., 2021 [26]	Simplified cEEG at six TTM sites using F3, P3, F4, P4, Cz reference, and Fz ground	TTM trial cEEG monitoring; 69% of ESE developed within 24 h	NR	Postanoxic ESE by cEEG	Periodic/rhythmic epileptiform discharges ≥ 1 Hz, continuously ≥ 90% of a 30 min period, or recurrent unequivocal electrographic seizures ≥ 10 s during a 30 min period	NR	Yes for unequivocal seizure criteria	All cEEG interpretations performed by an EEG specialist blinded to all clinical data
Admiraal et al., 2025 [27]	cEEG with at least 8 electrodes according to the 10–20 system plus reference and ground	cEEG started median 7.4 [5.8–9.2] h after CA	Central blinded review of consecutive 6 h epochs from 6 ± 3 h to 72 ± 3 h after CA	ACNS 2021 critical care EEG terminology	Not PSE-specific; prognostic EEG predictors	Early synchronous and late malignant patterns	Yes, ACNS 2021	One EEG specialist blinded to all clinical data reviewed each epoch
Rossetti et al., 2007 [28]	Portable digital EEG using 14 or 21 electrodes according to the 10–20 system	Median latency from admission 2 days	NR	Study-specific SE definition including PEDs and epileptiform burst-suppression	PSE defined as >5 min spontaneous or stimulus-induced repetitive/rhythmic focal or generalized spikes, sharp waves, spike-waves, or rhythmic waves evolving in amplitude, frequency, or field; PEDs and epileptiform burst-suppression also considered electrographic SE	PEDs/burst-suppression considered electrographic SE	NR	Retrospective review by EEG-certified author
Rittenberger et al., 2012 [29]	22-channel digital cEEG for first 48 h after resuscitation; minority had video cEEG	Median 9 [6–12] h from arrest to cEEG placement	First 48 h after resuscitation as standard monitoring	Standard definitions used for NCSE	NCSE defined as impaired consciousness with continuous electrographic seizure ≥ 30 min, recurrent electrographic seizures > 30 min, GPEDs ≥ 2.5 Hz for ≥30 min, or GPEDs ≥ 1 Hz for ≥30 min with evolution	NR	NR	EEGs interpreted clinically by board-certified neurologists; malignant cEEG files subsequently reviewed by two clinical neurophysiologists; outcome blinding not clearly reported
Shih et al., 2025 [30]	EEG data within first 5 days post-CA abstracted from clinical reports	Within 120 h	NR	ACNS-defined criteria within first 5 days post-CA	Unfavorable EEG included seizure, status epilepticus, myoclonic status epilepticus, GPDs, LPDs, or burst suppression	GPD *n* = 73/166; LPD *n* = 2/166; GRDA *n* = 7/166	NR	EEG/SSEP data were abstracted from clinical reports prepared by board-certified epileptologists or clinical neurophysiologists; no blinded central EEG rereview reported
De Stefano et al., 2025 [11]	cEEG or repetitive spot EEG; all available EEGs rereviewed	Definite/possible SE within 72 h after CA	NR	ACNS criteria for definite and possible SE	Definite electrographic SE: epileptiform discharges > 2.5 Hz or definite spatial/temporal evolution for ≥10 min or ≥20% of any 60 min recording period; electroclinical SE if clinical correlate time-locked or EEG/clinical improvement after ASM trial	Possible SE/IIC: periodic discharge or spike-wave > 1 to ≤2.5 Hz; or ≥0.5 to ≤1 Hz with plus modifier/fluctuation; or LRDA > 1 Hz with plus modifier/fluctuation, not qualifying as definite SE	Yes	All EEGs from three cohorts rereviewed by P.D.S. or N.G. blinded to outcome; controversies resolved by consensus

ACNS, American Clinical Neurophysiology Society; ASM, antiseizure medication; BIPD, bilateral independent periodic discharge; CA, cardiac arrest; cEEG, continuous electroencephalography; ED, epileptiform discharge; EEG, electroencephalography; ESE, electrographic status epilepticus; GPD, generalized periodic discharge; GPED, generalized periodic epileptiform discharge; GRDA, generalized rhythmic delta activity; ICU, intensive care unit; IIC, ictal–interictal continuum; ILAE, International League Against Epilepsy; LPD, lateralized periodic discharge; LRDA, lateralized rhythmic delta activity; NCSE, nonconvulsive status epilepticus; NR, not reported; PED, periodic epileptiform discharge; PLED, periodic lateralized epileptiform discharge; PSE, post-anoxic status epilepticus; RDA, rhythmic delta activity; rEEG, routine electroencephalography; ROSC, return of spontaneous circulation; RPP, rhythmic or periodic pattern; SE, status epilepticus; SSEP, somatosensory evoked potential; TTM, targeted temperature management.

**Table 4:** Treatment, electrographic response, and treatment-effect evidence.

Study	Design	Population/Arm	Treatment/Comparison	Key Outcomes/Response
Ruijter et al., 2022 [7]	Randomized treatment trial arm	Antiseizure-treatment	Protocolized antiseizure treatment strategy	Poor CPC 79/88 (89.8%); death 70/88 (79.5%); ≥48 h suppression 49/88 (55.7%)
		Control	Standard care/control	Poor CPC 77/84 (91.7%); death 69/84 (82.1%); ≥48 h suppression 2/83 (2.4%)
Hofmeijer et al., 2014 [23]	Observational/treatment response	Treated ESE	Unstandardized moderate AED/sedative treatment	Cessation/response 15/24 (62.5%)
De Stefano et al., 2025 [11]	Observational/treatment response	Whole definite/possible SE cohort	SE treatment per local/international stepwise recommendations	Cessation/response 120/274 (43.8%)
		Definite SE with <2 poor-outcome predictors	Guideline-recommended SE treatment and ASM dose intensity	Cessation/response 32/52 (61.5%)
Orav et al., 2023 [19]	Observational/treatment response	Post-hypoxic SE cohort	ASM treatment according to clinical practice	Cessation/response 45/52 (86.5%)
Lybeck et al., 2021 [26]	Observational/treatment response	ESE subgroup	Antiepileptic therapy exposure	NR
		No ESE subgroup	Antiepileptic therapy exposure	NR
Faro et al., 2019 [22]	Observational/treatment response	≥3 AED subgroup	High-intensity AED exposure	NR
Rittenberger et al., 2012 [29]	Observational/treatment response	NCSE	Local protocol: lorazepam bolus, phenytoin loading; levetiracetam/valproate next; midazolam/pentobarbital for refractory cases	NR

ASM, antiseizure medication; AED, antiepileptic drug (source-study terminology); CPC, Cerebral Performance Category; ESE, electrographic status epilepticus; NCSE, nonconvulsive status epilepticus; NR, not reported; SE, status epilepticus. Randomized treatment evidence and observational treatment-response evidence were not pooled because intervention exposure and electrographic response were confounded by indication, denominator selection, and overlap across source cohorts.

**Table 5:** GRADE certainty of evidence summary.

Outcome/Evidence Body	Studies (Participants)	Effect Estimate	Risk of Bias	Inconsistency	Indirectness	Imprecision	Publication Bias	Overall Certainty	Interpretation
Strict NCSE/ESE/PSE prevalence in non-overlapping EEG-monitored post-CA cohorts	4 studies; 457 patients; 112 events	22% (95% CI, 10–41%); PI, 4–64%	Not serious/serious *	Serious	Not serious	Serious	Not assessable	Low	NCSE/ESE/PSE is probably common, but the exact prevalence is uncertain across settings.
Strict NCSE/ESE/PSE prevalence including selected/historical/restricted cohorts	7 studies; 858 patients; 199 events	22% (95% CI, 16–31%); PI, 9–47%	Serious	Serious	Serious	Serious	Not assessable	Very low	Expanded estimates were directionally consistent but less generalizable because of selected denominators.
Broad electrographic seizure/status prevalence	5 studies; 1289 patients; 83 events	10% (95% CI, 2–33%); PI, 0–80%	Serious	Very serious	Serious	Very serious	Not assessable	Very low	The frequency of broader seizure/status constructs is highly uncertain.
Broad epileptiform/IIC pattern prevalence	3 studies; 968 patients; 293 events	29% (95% CI, 7–70%); PI, 2–92%	Serious	Serious	Very serious	Very serious	Not assessable	Very low	The estimate combines non-equivalent EEG constructs and should be considered exploratory.
Poor neurological outcome among patients with NCSE/ESE/PSE or related EEG-defined conditions	7 cohorts; 477 patients	91% (95% CI, 85–94%)	Serious	Not serious	Serious	Not serious	Not assessable	Low	Poor outcome is frequent, but causal interpretation is limited by WLST and baseline injury severity.
Mortality/death among patients with NCSE/ESE/PSE	6 cohorts; 213 patients	87% (95% CI, 78–93%)	Serious	Not serious	Serious	Not serious	Not assessable	Low	Mortality is high, but estimates are affected by outcome timing and WLST practices.
Association of SE/status versus no SE/status with poor neurological outcome	2 studies; 250 patients	OR, 18.22 (95% CI, 0.13–2570.33)	Serious	Not serious	Serious	Very serious	Not assessable	Very low	The association is directionally adverse but too imprecise for firm inference.
Protocolized suppression of rhythmic/periodic EEG activity versus standard care	1 RCT; 172 patients	Poor outcome: 89.8% vs. 91.7%; death: 79.5% vs. 82.1%; sustained EEG suppression: 55.7% vs. 2.4%	Serious/not serious †	Not applicable	Not serious for RPP trial population	Not serious	Not assessable	Moderate	Protocolized suppression improved EEG suppression but did not improve patient-important outcomes in an unselected RPP cohort.
Observational treatment-response/SE cessation evidence	3 main observational sources; 350+ patients across cohorts	SE cessation: 43.8–86.5% where reported; association with favourable outcome in selected cohorts	Very serious	Serious	Serious	Serious	Not assessable	Very low	Treatment-response associations are hypothesis-generating only because of confounding by indication and prognostic selection.

* Downgrading was driven mainly by heterogeneity and imprecision rather than uniform serious bias in all primary cohorts. † TELSTAR was downgraded because treatment was open-label, although endpoint assessment was blinded. CA, cardiac arrest; CI, confidence interval; EEG, electroencephalography; ESE, electrographic status epilepticus; IIC, ictal–interictal continuum; NCSE, nonconvulsive status epilepticus; PI, prediction interval; PSE, post-anoxic status epilepticus; RCT, randomized controlled trial; RPP, rhythmic or periodic pattern; SE, status epilepticus; WLST, withdrawal of life-sustaining therapy. For prevalence and absolute outcome-frequency estimates, certainty started at high and was downgraded according to GRADE domains. Thus, the primary prevalence estimate was downgraded one level for serious inconsistency and one level for serious imprecision, resulting in low certainty.

## Data Availability

The raw data supporting the conclusions of this article will be made available by the authors upon request.

## Supplementary Materials

The following supporting information can be downloaded at https://www.mdpi.com/article/10.3390/brainsci16090994/s1: Supplementary File S1: Completed PRISMA 2020 checklist. Supplementary File S2: Full database search strategies and search-results log. Supplementary File S3: Detailed risk-of-bias assessments using the JBI prevalence checklist, ROBINS-I, and RoB 2. Supplementary File S4: Study-level characteristics and prevalence data used for the quantitative analyses (worksheets: Characteristics and Prevalence). Supplementary File S5: R scripts used for statistical analyses and figure generation.
