# Supplementary material for: Nonconvulsive and Electrographic Status Epilepticus in Comatose Adult Survivors of Cardiac Arrest: A Systematic Review and Meta-Analysis of Prevalence, EEG-Based Diagnosis, Treatment, and Neurological Outcomes
Source: Brain Sci. 2026 Sep 19;16(9):994. doi: 10.3390/brainsci16090994 (PMC13604066; doi:10.3390/brainsci16090994)
Supplement: Supplementary file 1 [file brainsci-16-00994-s001.zip › Supplementary File S2 Full database search strategies and search-results log.pdf]

1. Full Search Strings by Database

1.1 PubMed.gov for PubMed / MEDLINE — Full String

```
("Heart Arrest"[Mesh] OR "cardiac arrest" OR "sudden cardiac arrest" OR
"out-of-hospital cardiac arrest" OR OHCA OR "in-hospital cardiac arrest" OR IHCA OR
"post cardiac arrest" OR "post-cardiac arrest" OR "post-resuscitation" OR ROSC OR
"return of spontaneous circulation")
AND
("Status Epilepticus"[Mesh] OR "nonconvulsive status epilepticus" OR
"non-convulsive status epilepticus" OR NCSE OR "electrographic status epilepticus" OR
"post-anoxic status epilepticus" OR "postanoxic status epilepticus" OR
"electrographic seizure*" OR "nonconvulsive seizure*" OR "non-convulsive seizure*" OR
"ictal-interictal continuum" OR "ictal interictal continuum" OR "periodic discharge*" OR
"generalized periodic discharge*" OR GPD OR "lateralized periodic discharge*" OR LPD OR
"rhythmic delta activity" OR "rhythmic periodic pattern*" OR epileptiform)
```

1.2 MEDLINE Core database via Clarivate

```
("cardiac arrest" OR "sudden cardiac arrest"
OR "out-of-hospital cardiac arrest" OR OHCA
OR "in-hospital cardiac arrest" OR IHCA
OR "post cardiac arrest" OR "post-resuscitation"
OR ROSC OR "return of spontaneous circulation")
AND
("nonconvulsive status epilepticus" OR "non-convulsive status epilepticus" OR NCSE
OR "electrographic status epilepticus"
OR "post-anoxic status epilepticus" OR "postanoxic status epilepticus"
OR "electrographic seizure*" OR "nonconvulsive seizure*"
OR "ictal-interictal continuum"
OR "periodic discharge*" OR GPD OR LPD
OR epileptiform)
```

1.3 Web of Science Core Collection via Clarivate

```
TS= (("cardiac arrest" OR "sudden cardiac arrest"
OR "out-of-hospital cardiac arrest" OR OHCA
```

```
OR "in-hospital cardiac arrest" OR IHCA
OR "post cardiac arrest" OR "post-resuscitation"
OR ROSC OR "return of spontaneous circulation")
AND
TS= ("nonconvulsive status epilepticus" OR "non-convulsive status epilepticus" OR NCSE
OR "electrographic status epilepticus"
OR "post-anoxic status epilepticus" OR "postanoxic status epilepticus"
OR "electrographic seizure*" OR "nonconvulsive seizure*"
OR "ictal-interictal continuum"
OR "periodic discharge*" OR GPD OR LPD
OR epileptiform))
```

1.4 Elsevier Scopus

```
TITLE-ABS-KEY(
("cardiac arrest" OR "sudden cardiac arrest"
OR "out-of-hospital cardiac arrest" OR OHCA
OR "in-hospital cardiac arrest" OR IHCA
OR "post cardiac arrest" OR "post-cardiac arrest"
OR "post-resuscitation" OR ROSC OR "return of spontaneous circulation")
AND
TITLE-ABS-KEY("nonconvulsive status epilepticus" OR "non-convulsive status epilepticus" OR "NCSE"
OR "electrographic status epilepticus"
OR "post-anoxic status epilepticus" OR "postanoxic status epilepticus"
OR "electrographic seizure*" OR "nonconvulsive seizure*"
OR "non-convulsive seizure*"
OR "ictal-interictal continuum" OR "ictal interictal continuum"
OR "periodic discharge*" OR "generalized periodic discharge*" OR GPD
OR "lateralized periodic discharge*" OR LPD
OR "rhythmic delta activity" OR "rhythmic periodic pattern*"
OR epileptiform)
)
```

1.5 Cochrane Library for CENTRAL

```
("cardiac arrest" OR OHCA OR IHCA OR ROSC OR "post-resuscitation")
AND
```

("nonconvulsive status epilepticus" OR NCSE OR
"electrographic status epilepticus" OR
"post-anoxic status epilepticus" OR
"electrographic seizure*" OR "nonconvulsive seizure*" OR
"ictal-interictal continuum" OR epileptiform)

2. Search Results Log

Database	Records Retrieved
PubMed / MEDLINE	347
MEDLINE Core	222
Cochrane CENTRAL	11
Web of Science	393
Scopus	272
**Total (before deduplication)**	1245
Duplicates removed	666
**Total (after deduplication)**	579
