# Supplementary material for: Nonconvulsive and Electrographic Status Epilepticus in Comatose Adult Survivors of Cardiac Arrest: A Systematic Review and Meta-Analysis of Prevalence, EEG-Based Diagnosis, Treatment, and Neurological Outcomes
Source: Brain Sci. 2026 Sep 19;16(9):994. doi: 10.3390/brainsci16090994 (PMC13604066; doi:10.3390/brainsci16090994)
Supplement: Supplementary file 1 [file brainsci-16-00994-s001.zip › Supplementary File S3 Detailed risk-of-bias assessments using the JBI prevalence checklist, ROBINS-I, and RoB 2.pdf]

## Supplementary risk-of-bias tables

Supplementary Table S1. JBI prevalence checklist appraisal for observational/descriptive studies.

| **Study** | **Q1** | **Q2** | **Q3** | **Q4** | **Q5** | **Q6** | **Q7** | **Q8** | **Q9** | **Overall judgement** |
|---|---|---|---|---|---|---|---|---|---|---|
| Pelle et al., 2025 | Y | Y | Y | Y | Y | Y | Y | Y | Y | Moderate |
| Dragancea et al., 2015 | Y | Y | Y | Y | Y | Y | Y | Y | Y | Moderate |
| Backman et al., 2017 | N | N | Y | Y | Y | Y | Y | Y | Y | Serious |
| Peluso et al., 2024 | N | N | Y | Y | Y | Y | U | Y | Y | Serious |
| Orav et al., 2023 | N | N | Y | Y | Y | Y | Y | Y | Y | Serious |
| Faro et al., 2019 | Y | U | Y | Y | U | Y | U | Y | Y | Serious |
| Mani et al., 2012 | Y | Y | N | Y | Y | Y | Y | Y | Y | Moderate |
| Admiraal et al., 2025 (PSE profile) | Y | Y | Y | Y | Y | Y | Y | Y | Y | Moderate |
| Lybeck et al., 2021 | U | U | Y | Y | Y | Y | Y | Y | Y | Moderate |
| Admiraal et al., 2025 (EEG prognostic patterns) | Y | Y | Y | Y | Y | Y | Y | Y | Y | Moderate |
| Rossetti et al., 2007 | U | N | Y | Y | N | U | U | Y | Y | Serious |
| Rittenberger et al., 2012 | Y | Y | Y | Y | Y | Y | U | Y | Y | Moderate |
| Shih et al., 2025 | Y | U | Y | Y | N | Y | U | Y | Y | Serious |

JBI prevalence checklist items: Q1 = sample frame appropriate to address the target population; Q2 = participants sampled appropriately; Q3 = adequate sample size; Q4 = subjects and setting described in detail; Q5 = data analysis conducted with sufficient coverage of the identified sample; Q6 = valid methods used for identification of the condition; Q7 = condition measured in a standard and reliable way for all participants; Q8 = appropriate statistical analysis; Q9 = adequate response rate or appropriate handling of low response. Response options: Y = yes; N = no; U = unclear; NA = not applicable.

Supplementary Table S2. ROBINS-I appraisal for non-randomized treatment-effect studies.

| Study | Confounding | Selection of participants | Classification of interventions | Deviations from intended interventions | Missing data | Measurement of outcomes | Selection of reported result | Overall judgement |
|---|---|---|---|---|---|---|---|---|
| Hofmeijer et al., 2014 | Serious/Critical | Moderate | Moderate | Serious | Moderate | Low | Moderate | Serious/Critical |
| De Stefano et al., 2025 | Serious | Moderate | Moderate | Moderate/Serious | Moderate | Low | Moderate | Serious for treatment effect; Moderate for prognosis |

Supplementary Table S3. RoB 2 appraisal for randomized studies

| Study | Randomization process | Deviations from intended interventions | Missing outcome data | Measurement of outcome | Selection of reported result | Overall judgement |
|---|---|---|---|---|---|---|
| Ruijter et al., 2022 | Low | Some concerns | Low | Low | Low | Some concerns |
| Urbano et al., 2022 | Low | Some concerns | Low/Moderate | Low | Moderate | Some concerns/Moderate |
